# Morphological and stage-specific transcriptome analyses reveal distinct regulatory programs underlying yam (*Dioscorea alata* L.) bulbil growth

**DOI:** 10.1093/jxb/erz552

**Published:** 2019-12-13

**Authors:** Zhi-Gang Wu, Wu Jiang, Zheng-Ming Tao, Xiao-Jun Pan, Wen-Hui Yu, Hui-Lian Huang

**Affiliations:** 1 Key Laboratory for Plant Genetic Improvement, Institute of Subtropical Crops, Zhejiang Academy of Agricultural Sciences, Wenzhou, China; 2 School of Pharmacy, Wenzhou Medical University, Wenzhou, China; 3 Quzhou Academy of Agricultural Sciences, Quzhou, China; 4 Key Laboratory of Modern Preparation of Traditional Chinese Medicine, Jiangxi University of Traditional Chinese Medicine, Nanchang, China; 5 RWTH Aachen University, Germany

**Keywords:** Bulbil, genetic regulation, growth, transcriptome, phytohormone, yam (*Dioscorea alata* L)

## Abstract

In yam (*Dioscorea* spp) species, bulbils at leaf axils are the most striking species-specific axillary structure and exhibit important ecological niches. Genetic regulation underlying bulbil growth remains largely unclear so far. Here, we characterize yam (*Dioscorea alata* L.) bulbil development using histological analysis, and perform full transcriptional profiling on key developmental stages together with phytohormone analyses. Using the stage-specific scoring algorithm, we have identified 3451 stage-specifically expressed genes that exhibit a tight link between major transcriptional changes and stages. Co-expressed gene clusters revealed an obvious over-representation of genes associated with cell division and expansion at the initiation stage of bulbils (T1). Transcriptional changes of hormone-related genes highly coincided with hormone levels, indicating that bulbil initiation and growth are coordinately controlled by multiple phytohormones. In particular, localized auxin is transiently required to trigger bulbil initiation, and be further depleted or exported from bulbils to promote growth by up-regulation of genes involved in auxinconjugation and efflux. The sharp increase in supply of sucrose and an enhanced trehalose-6-phophate pathway at T1 were observed, suggesting that sucrose probably functions as a key signal and promotes bulbil initiation. Analysis of the expression of transcription factors (TFs) predicated 149 TFs as stage-specifically expressed; several T1-specific TFs (from Aux/IAA, E2F, MYB, and bHLH families) have been shown to play key roles in triggering bulbil formation. Together, our work provides a crucial angle for in-depth understanding of the molecular programs underlying yam’s unique bulbil development processes. Stage-specific gene sets can be queried to obtain key candidates regulating bulbil growth, serving as valuable resources for further functional research.

## Teaser

Phytohormones and sucrose coordinately control bulbil initiation, and provide forward signaling to genes that maintain the vitality of cell proliferation and expansion during growth.

## Introduction

Plants exhibit a high degree of plasticity in axillary forms including dormant axillary buds, tillers, vegetative branches, or other specific axillary structures (Li *et al.*, 2003; Evers *et al.*, 2011). In some circumstances, plants evolve unique aerial bulbils derived from the growth of meristems in the axils of leaves or bracts, in response to the ecological or evolutionary niches in which the plant grows (Law *et al.*, 1983; Szarek *et al.*, 1996; Ronsheim and Bever, 2000; Deng *et al.*, 2013). In particular, the bulbils released from the mother plants can grow into new individuals in the next growth season, and offer a failsafe strategy of asexual reproduction when seeds are absent under harsh environmental conditions (Arizaga and Ezcurra, 2002; Walck *et al.*, 2010).

Bulbils are rarely observed in nature, but are generally seen in several plant populations from *Agave tequilana*, *Titanotrichum oldhamii*, *Polygonum viviparum*, *Lilium lancifolium*, *Poa alpina*, and numerous members of *Dioscorea* (Law *et al.*, 1983; Murty and Purnima, 1983; Szarek *et al.*, 1996; Wang and Cronk, 2003; Steiner *et al.*, 2012; Yang *et al.*, 2017). The production of these species-specific structures and their developmental patterns are distinct. In *Agave* and *Titanotrichum* plants, the floral meristems develop into numerous bulbils by repeated subdivision when the flowers fade away; this type of bulbil occurs on the sides of the inflorescence or pedicel (Wang and Cronk, 2003; Sandoval *et al.*, 2012). In some cases, bulbils are also derived from the adjacent proximal meristems (Moody *et al.*, 1999). For most bulbil-producing plants, such as *Dioscorea* and *Lilium* species, bulbils similar to buds are generated from axillary meristems (AMs) at the axil when apical dominance ceases (Murty and Purnima, 1983; Yang *et al.*, 2017).

Little available information has been documented so far to explain the regulation processes of bulbil initiation and growth. Consistent with buds originated from AMs, bulbil growth involves two developmental stages, namely (i) the formation of new AMs induced by bulbil primordia; and (ii) continuous cell division of AMs for establishing bulbil form (Li *et al.*, 2012; Yang *et al.*, 2017; Wang and Jiao, 2018). Limited evidence has demonstrated that the fate of early bulbil formation, whether to grow or remain dormant, is strongly dependent on environmental factors, phytohormone signals, and genetic factors (Fan and Yang, 2009; Steiner *et al.*, 2012; Abraham-Juárez *et al*., 2015). Bulbil-producing plants of *P. alpina* often occur in high altitude populations, and remain strongly vigorous (Steiner *et al.*, 2012). *Titanotrichum* plants easily induce the transition from the flower to bulbil meristems when exposed to short-day conditions (autumn); and individuals under dense shade can produce more bulbils even under full light (Wang and Cronk, 2003). More importantly, plant hormones play crucial roles in regulation of bulbil formation at the early stage. In *Agave* plants, the reduction of auxin by direct removal of flower buds induces bulbil initiation, whereas exogenous application of auxin on cut pedicle tissue represses bulbil growth (Abraham-Juárez *et al*., 2015). Similarly, the short-term accumulation of auxin in upper axils activates bulbil initiation in *Lilium* species (Yang *et al.*, 2017). Elevated gibberellin (GA) significantly increases bulbil yield of Chinese yam by exogenous application (Kim *et al.*, 2003). Several genetic studies have revealed that bulbil initiation is regulated by multiple auxin-mediated transcription factors (TFs), such as KNOX, MADS, and LEAFY (Wang *et al.*, 2004; Abraham-Juárez *et al.*, 2010; Sandoval *et al.*, 2012). Alternatively, the PIN proteins localized in the pedicel function by modulating auxin gradients in *A. tequilana*, and play a central role in the initial stage of bulbil formation (Abraham-Juárez *et al*., 2015). A gesneriaceae-*FLORI*-*GAULA* (GFLO) gene in *T. oldhamii* that switches the process of flowering to bulbil production, has been identified as a key candidate regulating bulbil formation (Wang *et al.*, 2004). Whereas little is known in terms of understanding the regulatory mechanisms of bulbil growth, there has been a massive increase in understanding of axillary bud growth over the past 30 years (Chatfield *et al.*, 2000; Wang and Jiao, 2018; Chabikwa *et al.*, 2019). Two widely accepted theories, auxin transport canalization (ATC) and the second messenger (SM) models, have been proposed to describe the mechanisms regulating bud growth (Domagalska and Leyser, 2011). Central to the ATC model is that auxin from the shoot apex inhibits the outgrowth of buds, and must be depleted to activate expansion of new AMs, in which the polar auxin transport (PAT) stream is essential and driven by PIN efflux proteins (Balla *et al.*, 2011). The SM model contends that auxin does not enter the bud, and regulates the production of axillary buds by second messengers, which directly move into the bud to control its activity (Domagalska and Leyser, 2011). These messengers include the hormones cytokinin (CK) and abscisic acid (ABA) (Yao and Finlayson, 2015; Roman *et al.*, 2016).

Yams (*Dioscorea* spp) represent one of the most primitive angiosperms and differentiated species (Burkill, 1960). In >600 members recorded in this genus, the most striking axillary plasticity is the occurrence of bulbils that are termed aerial tubers or minor storage organs (Wickham *et al.*, 1982; Murty and Purnima, 1983). Ecologically, this structure, initiated at leaf axils, enables the plants to spread rapidly and engulf the native vegetation (Mizuki and Takahash, 2009). Yam bulbils also serve as an effective means for vegetative reproduction, and are often dormant and will germinate when they drop to the ground in the following season (Main *et al.*, 2006). In many yam species, aerial bulbils are very common and have been produced widely as food or for pharmaceutical uses (Asiedu and Sartie, 2010). Despite their importance in terms of commercial values and ecological plasticity, the gene regulatory programs throughout bulbil growth still remain largely unclear. Here, we selected a typical yam species (*D. alata* Linn.) and combined morphological analysis, and phytohormone- and stage-specific expression profiles, to uncover the regulatory events underlying bulbil growth. We established a regulatory model, and reported the key roles of auxin, CK, and sucrose as signals during bulbil initiation. We also identified stage-specifically expressed gene sets associated with regulation of cell division, proliferation, and expansion underlying early bulbil formation, which provide valuable genomic resources for further functional analysis.

## Materials and methods

### Plant materials

Homogeneous seedlings of yam (*D. alata*) were asexually propagated using severed tubers (100–150 g per plant) from the same mother plant. The seedlings were grown in Wenzhou botanical garden (Wenzhou city, Zhejiang province, China) under standard conditions. The bulbils were seen in the axil of leaf primordia after 130 d growth. Four staged samples of bulbil were harvested at 1 day after bulbil emergence (1 DAE) termed the initiation stage (labeled T1), early stage (7 DAE, T2), middle stage (15 DAE, T3), and mature stage (35 DAE, T4). Five plants were collected for each bulbil type.

### Histological analyses of bulbil development

Bulbils across the four investigated stages were fixed in FAA solution (70% ethanol:formalin:acetic acid, 90:5:5) for 24 h at 4 °C. The fixed bulbils were dehydrated through a graded ethanol series and embedded in paraffin, as described previously (Xing *et al.*, 2010). Longitudinal sections of 10 μm were prepared with a rotary microtome and stained in safranin–alcian green according to standard histological procedures (Gutmann,1995). Stained sections were observed and documented under an OLYMPUS BX60 light microscope (Olympus, Japan).

### Determination of sucrose content

A 20 mg aliquot of bulbils was ground in liquid nitrogen and extracted with 80% ethanol for 40 min at 60 °C in cap-sealed tubes using 1 ml per 200 mg sample. The extraction was carried out twice with the same conditions. The combined suspension was centrifuged at 10 000 *g* for 10 min at 4 °C. The supernatant was analyzed and sucrose contents were determined with HPLC-evaporative light scattering detector (ELSD) analysis (Agilent 1200) as described previously (Ma *et al.*, 2014). Five independent experiments were carried out for each bulbil type.

### Phytohormone metabolic profiles

CK metabolites (zeatin and dihydrozeatin), free indoleacetic acid (IAA), and ABA were detected by using an LC-ESI-MS/MS system (HPLC, Shim-pack UFLC SHIMADZUCBM30A system; MS, Applied Biosystems 6500 Triple Quadrupole) (Šimura *et al.*, 2018). A 50 mg (FW) portion of bulbils was homogenized in liquid nitrogen and extracted with methanol:water:formic acid (15:4:1, v/v/v). The extracts were further dried under a nitrogen gas stream and reconstituted in 80% methanol (v/v). The treated extracts were separated using an ACQUITY UPLC HSS T3 C18 (1.8 μm, 2.1 mm×100 mm) (Waters). Analytes were eluted by a binary gradient phase constituted of ice-cold acetonitrile (with 0.01% v/v formic acid) (A) and water (with 0.01% v/v formic acid) (B), with a flow rate of 0.35 ml min^–1^. The gradient programs were as follows: 0–1 min, 5% (v/v) A; 1–8 min, 5–95% (v/v) A; 8–9 min, 95% (v/v) A; 9–12 min, 5% (v/v) A. Levels of IAA, zeatin, dihydrozeatin, and ABA were determined by Metware (http://www.metware.cn/) based on the AB Sciex QTRAP 6500 LC-MS/MS platform. Three replicates of each assay were performed.

### RNA sequencing and bioinformatics analyses

Total RNA of each sample was isolated using TRIzol reagent (Invitrogen) and purified using DNase I (TURBO DNase, Ambion, USA). The integrity of RNA (RIN >8.5) was detected using a Bioanalyzer 2100 (Agilent Technologies, Santa Clara, CA, USA). RNA sequencing (RNA-seq) libraries were prepared using the cDNA Synthesis kit (Illumina Inc., San Diego, CA, USA) following the standard Illumina preparation protocol. Paired-end sequencing (2×150 bp) was conducted with Illumina HiSeq 2500 (Illumina Inc.) by the Biomarker Biotechnology Corporation (Beijing, China). Three independent biological replicates were analyzed for each bulbil type. The raw reads were refined by removing reads with only adaptor and unknown nucleotides >10%, and low-quality reads with the average phred quality score <30. The clean reads were used for robust *de novo* assembly of a set of transcriptomes using the Trinity software package (version R2013-02-25) (Haas *et al.*, 2013).

The expression abundance of each transcript was estimated by calculating the FPKM (fragments per kilobase of exon per million fragments mapped) value using TopHat (version 2.0.8) (Trapnell *et al.*, 2012). Based on overall expression profiles, correlation of three biological replicates was calculated to assess the reliability of sample collection using the cor R package. Differentially expressed genes (DEGs) between differential bulbil types were identified using the Edge statistical test in terms of the following criteria: false discovery rate (FDR) <0.01, and an absolute expression foldchange (FC) ≥2 for a given gene between any comparisons (Robinson *et al.*, 2010). We annotated biological function for DEGs using NR public databases according to BLASTX analysis with a cut-off E-value of 10^−5^. GO slim was conducted to obtain Gene Ontology (GO) annotations using Blast2GO (Conesa *et al.*, 2005). To obtain enriched slims, we further performed GO term enrichment analysis using the algorithm and the Kolmogorov–Smirnov (KS) test (*P*-value≤0.001) in the R package topGO (Alexa *et al.*, 2006). Based on all expressed genes, principal component analysis (PCA) was carried out to explain the relatedness among all samples. For all DEGs, we performed hierarchical cluster analysis (HCA) to present gene expression patterns using the pheatmap library in R software. In addition, TFs were identified in terms of the Arabidopsis transcription factor database (Perez-Rodriguez *et al*., 2010).

### Identification of stage-specific (SS) gene sets

The genes specifically expressed in each stage type were identified using an SS scoring algorithm based on Zhan *et al.* (2015). For a given gene *i*, its expression values in four stages are described as *E*_*i*_=$(E_{1}^{i}$+$E_{2}^{i}$+$E_{3}^{i}$+$E_{4}^{i}$), and the SS score of this gene in stage *j* is defined as follows, SS(*i*, *j*)=1-$\frac{Max\;E_{k}^{i}}{E_{j}^{i}}$, where 1≤*k*≤4, *k*≠*j*. Therefore, SS scores range from 0 to 1, and genes with higher SS scores at a particular stage are likely to be more specifically expressed uding that stage. We here defined genes with an SS score >0.3 as being specifically expressed in corresponding stages.

### Validation of genes using quantitative RT–PCR

Quantitative reverse transcription–PCR (qRT–PCR) was performed to verify transcriptome profiles with 20 selected genes. Three biological replicates were carried out for each selected gene. The PCR amplification primers for selected genes were designed with Primer 3 software, and sequences are listed in Supplementary Table S1 at *JXB* online. RNA was extracted as described above. The cDNA was prepared with SuperScript III Reverse transcriptase (Invitrogen) following the manufacturer’s protocol on 1μg of RNA. The qRT–PCR amplification was run in an ABI 7500 HT (Life Technology) with SYBR Green I Master Mix (TaKaRa), and the reaction mixture and program were carried out as described in our previous work (Wu *et al*., 2015). Quantification of gene expression was normalized using the EF-1a gene (accession no. JF825419) as an internal control, and counted according to the 2^−ΔΔCt^ method (Livak and Schmittgen, 2001).

### RNA *in situ* hybridization

RNA *in situ* hybridization was performed using the method described by Siciliano *et al* (2007). Bulbils were fixed in FAA and embedded in paraffin as described above. Gene-specific fragments for probe synthesis were amplified by PCR using designed primers: 5'-GAAGAGCACCATGCTGTGAG-3' and 5'-TAATACGACTCACTATAGGGCCACATCTCAGCAATCCAG-3' for MYB (Gene ID: c126446.graph-c0), 5'-TGGAGAGCCTTTGATCGGTT-3' and 5'-TAATACGACTCACTATAGGGCCACTGCTCTAAACGAAGG-3' for WRKY (c125026.graph_c1), and 5'-AGTGCATTACCTCTGCCGGA-3' and 5'-TAATACGACTCACTATAGGTACCTGGCAATTCCCAAGGA-3' for NAC (c116834.graph_c0). The resulting PCR fragments were used as templates for synthesis of digoxigenin (DIG)-labeled antisense and sense riboprobes with the T7/SP6 riboprobe and a DIG-RNA labeling mix (Roche). Sections (8–10 μm) were treated with 1 μg ml^−1^ proteinase K for 30 min at 37°C, and then washed under stringent conditions as described previously (Hsu *et al.*, 2015).

## Results

### Bulbil morphology during its developmental stages

**Fig. 1.**
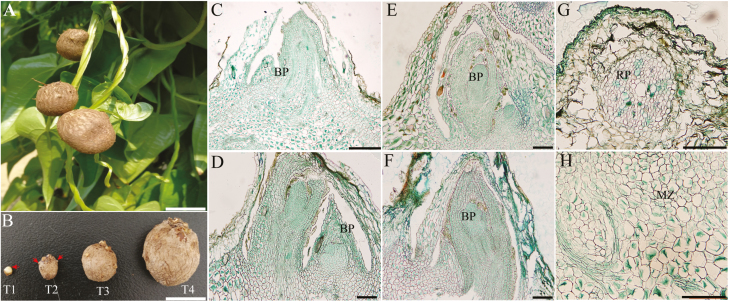
Morphology of the bulbil during key developmental stages. (A) Bulbil phenotype. (B) Photographs of the bulbil at the initiation (T1), early (T2), middle (T3), and mature stages (T4). (C–F) Paraffin sections of bulbils at T1 (C), T2 (D), T3 (E), and T4 (F) stages. The images show the zone of the junction region between the bulbil and axil. (G) Showing root primordia (RP). (H) Showing the meristematic zone (MZ). The arrows in T1 and T2 show the BP and root primordia, respectively. Scale bars (A and B), 1 cm; (C), 500 µm; (D–H), 200 µm.

Yam bulbils occur naturally on the leaf axil when the main apex stops growing (Fig. 1A). To characterize the developmental process of bulbils in detail, we designed four growing sequences based on our observations and important literature (Murty and Purnima, 1983), namely the initiation (T1), early (T2), middle (T3), and mature (T4) stages of bulbil formation (Fig. 1B). At the T1 stage, the cells of 2–3 layers below the leaf axil have undergone periclinal and anticlinal divisions, and developed into a hump-like meristematic tissue that is termed the bulbil primordium (BP) (Fig.1C). The BP at this stage was pivotal for subsequent bulbil outgrowth and still remained partly differentiated At the same time, a dome-shaped structure was visible in the leaf axil. At the T2 stage, the cells from the BP meristematic zone (Fig. 1H) became highly meristematic and showed successive cell division and enlargement (Fig. 1D), and ultimately formed the young bulbil. The young bulbil was shaped like a spinning top, and the root primordium (RP) was seen in the cortical region at this stage (Fig. 1G). At the next stage (T3), the size of the bulbil had enlarged rapidly, as the meristem in the central region of the bulbil continuously widened (Fig. 1E). The increased cell numbers and enlarged volume by filling starch grains resulted in quasi-round bulbils. The bulbil at this stage had a distinct peripheral region covered by several rough epidermal layers (Fig. 1B). At the T4 stage, the activity of meristematic tissue was mostly depleted (Fig. 1F) and mature bulbils reached 1.2–3.0 cm in diameter. Multiple RPs had emerged on the bulbil surface, which enable bulbils to spread rapidly in the next season. Taken these findings together, bulbil morphology was distinct at the four developmental stages.

### Global RNA-seq analysis of yam bulbils during development

Given the histological differences between bulbil stage types, we collected three biological replicates of each type for the preparation of RNA-seq libraries. All raw reads obtained by RNA-seq have been submitted to the SRA database under accession number SRP152752. High quality RNA-Seq data were obtained for each sample, with 49–66 million paired-end reads (Supplementary Table S2). The *de novo* assembly generated 199 270 transcripts; ~36% of transcripts were in the size range 200–500 bp (Supplementary Table S3). The homologous transcripts were further clustered with >95% similarity, and generated 97 956 unigenes with a total length of 79 527036 bp and an average length of 812 bp. Read counts per gene were calculated and normalized as FPKM (Trapnell *et al.*, 2012). An expressed gene was required to have an FPKM of >1.0 in at least one sequenced sample.

**Fig. 2.**
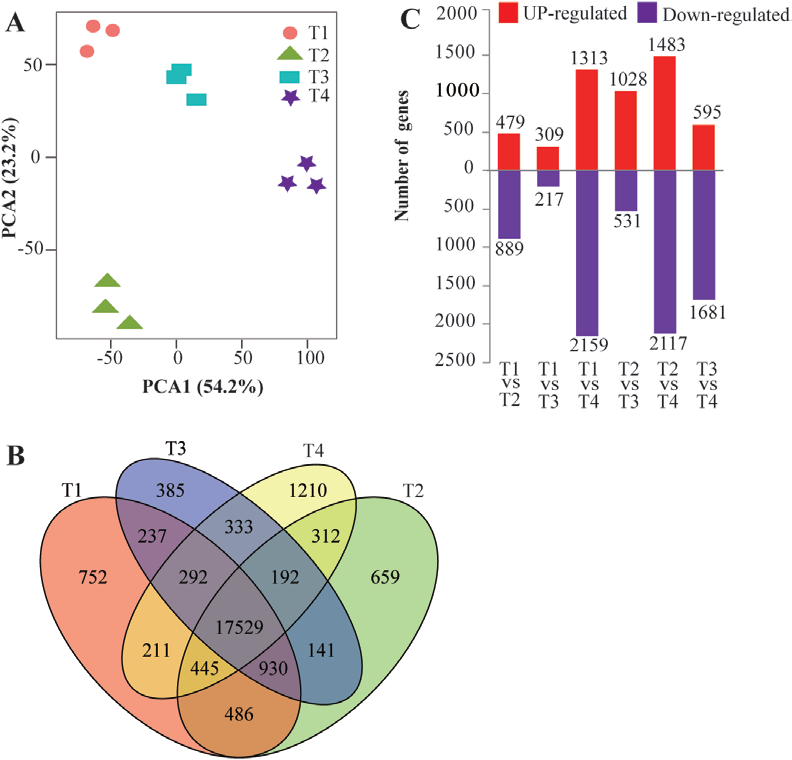
Gene expression profiles during bulbil growth. (A) Principal component analysis for 12 bulbil types showing the four stage-specific groups based on all gene expression profiles. (B) Venn diagram showing the numbers of unique and overlapping expressed genes in bulbils among different developmental stages (T1–T4). (C) Comparison of the number of up- and down-regulated genes between different stages.

PCA visualized four stage-specific clusters, with the first two components explaining 77.4% of the total variance, and revealed distinct mRNA populations between different bulbil types (Fig. 2A). Next, we observed that 752 (in T1), 659 (T2), 385 (T3), and 1210 (T4) genes had highly stage-specific expression patterns, but most of the genes (17 529) were shared in bulbils at all stages (Fig. 2B). Meanwhile, we assessed gene expression profiles between biological triplicates and they were highly correlated (*R*^2^ >0.83), (Supplementary Fig. S1) indicating that bulbils from the same stage were well collected, avoiding the variation of samples from the same stage.

To confirm the reliability of RNA-seq, we also performed a more rigorous expression measure for 20 selected genes using qRT–PCR analysis. We revealed a good agreement with a high linear correlation (*R*^2^ >0.80; see Supplementary Fig. S2) between RNA-seq and qRT–PCR technologies, suggesting the reliability of RNA-seq analyses.

Using a threshold value of FC >2 in expression difference and FDR <0.01 for filtering DEGs, we identified a total of 6112 DEGs in a least one comparison (Fig. 2C; Supplementary Table S4). These results represented substantial differences in gene expression profiles across bulbil growth. HCA using all DEGs revealed four discrete clusters corresponding to four time points (Supplementary Fig. S3), which constitute distinct gene sets, highlighting the specialized nature of different bulbil types, suggesting a tight linkage of DEGs with bulbil growth responses. Furthermore, GO enrichment analysis for DEGs indicated that GO terms including regulation of cell cycle, regulation of DNA replication, cell proliferation, and protein kinase activity, are strikingly enriched (corrected *P*-value <0.001) (Supplementary Table S5; Supplementary Fig. S4). Several significantly enriched KEGG (Kyoto Encyclopedia of Genes and Genomes) pathways were suggested to be linked to starch and sucrose metabolism, and plant hormone signal transduction (Supplementary Table S6; Supplementary Fig. S5).

### Stage-specific gene clusters contributing to bulbil initiation and growth

**Fig. 3.**
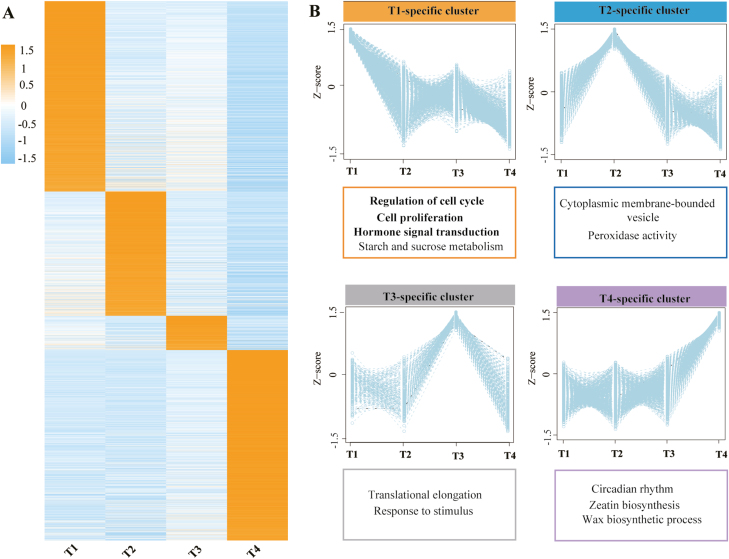
Stage-specific gene clusters. (A) Heat map of scaled FPKM values of 3451 stage-specific genes (with SS score >0.3) during bulbil growth. (B) Average expression profiles of each gene cluster and its corresponding enriched GO functional category. The sets of stage-specific genes are available in Supplementary Table S7.

Our aim in this study was to identify genes associated with yam bulbil growth from the different stages, especially for genes promoting bulbil initiation at the early stage. Therefore, we further narrowed the DEG set and identified sets of tissue-specifically expressed genes through the SS scoring algorithm (see the Materials and methods). In this analysis, we identified a total of 3451 SS genes (Fig. 3A; Supplementary TableS7). We found that the numbers of detected SS genes are obviouslydifferent among the four stage types. T1 and T4 types had the most SS genes, with 1218 genes being identified as stage specific at the two stages. In contrast, the T3 type had the lowest number of SS genes (220). These SS genes exhibited consistent expression patterns and peak abundances in the corresponding specific stages (Fig. 3B). We also found that most SS genes are over-represented in these enriched GO terms and KEGG pathways for all DEGs (Fig. 3B), suggesting that these SS genes may be important candidates for regulating bulbil growth. Accordingly, we classified SS genes into these enriched GO terms and pathways to elucidate their functions.

### The T1-specific cluster highly expressed a large number of genes associated with cell cycle and proliferation

**Fig. 4.**
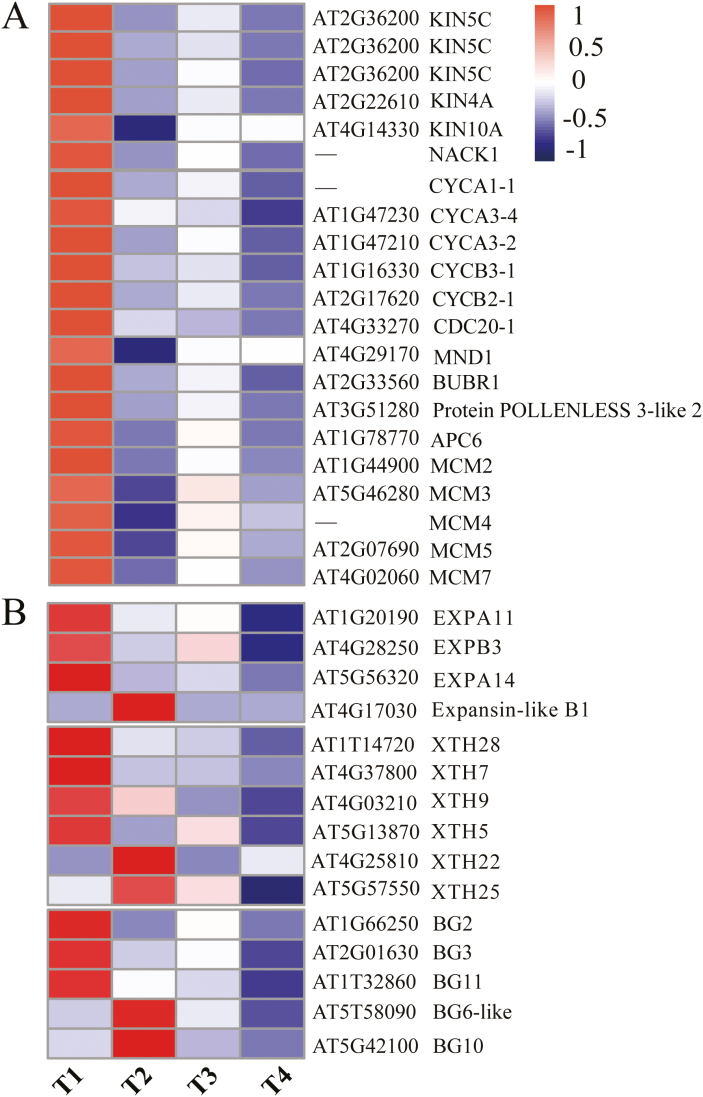
Heat maps of gene sets involved in the cell cycle and proliferation (A), and cell wall expansion (B). The gene expression levels are standardized into *Z*-scores and colored in red and blue for high and low expression, respectively. Gene names (shown on the right) are described in detail in Supplementary Dataset S1.

Interestingly, GO terms including regulation of the cell cycle, cytokinesis by cell plate formation, and cell proliferation were highly co-enriched in the T1-specific gene cluster (Fig. 3B), which hints at the co-regulation of genes coupled with these terms during transcriptomic responses to bulbil initiation. To better understand the control, we screened for SS genes involved in these enriched terms and detected 22 marker genes (Fig. 4A; Supplementary Dataset S1). These genes included seven genes encoding multiple kinesin-related proteins (three homologs of KIN5C, KIN4A, KIN10A, and NACK1) and a TIO kinase (AT1G50240), which are required to support cell plate growth in meristem tissue (Oh *et al.*, 2012; Tan *et al.*, 2019). Ten genes involved in the regulation of cell cycle or division were also observed, including genes encoding A/B-type cyclin proteins (CYCA1-1, CYCA3-2, CYCA3-4, CYCB2-1, and CYCB3-1), and multiple proteins (MND1, BUBR1, APC6, and *POLLENLESS 3-like 2* protein) that control progression through mitosis in the G_1_/2 phase of the cell cycle (Devitt and Stafstrom, 1995; Berckmans and Veylder, 2009).

In addition, we found five members of DNA replication licensing factors (MCM2, MCM3, MCM4, MCM5, and MCM7), which play crucial roles in the control of cell proliferation processes required for lateral meristem formation (Ni *et al.*, 2009). Unexceptionally, these genes were consistently induced and exhibited the highest expression at the initial stage of bulbil growth (T1) (Fig. 4A). For instance, as compared with the T2 stage, the transcript accumulation levels for the representative genes KIN5C (c132756.graph_c0), CYCA3-4 (c122639.graph_c0), and MCM4 (c105199.graph_c0) were up-regulated 4.8-, 2-, and 8-fold, respectively. Taken together, the trend in accumulation of cell cycle- and proliferation-related genes performed a quality control function to produce the large number of new cells required on the flanks of the stem and to ultimately produce the obvious visible bulbil meristem (Devitt and Stafstrom, 1995).

### Genes related to cell wall modification were induced at the early stage of bulbil growth

Cell wall extension, as a secondary event followed by cell division, plays a crucial regulatory role in inducing early primordium initiation in meristems and determining the size and shape of cell- and plant-specific organs (Chebli and Geitmann, 2017). We here searched known genes involved in modification of the cell wall network through expansion and loosening, and identified 15 SS genes in this process (Supplementary Dataset S1). These genes included four EXPANSINS (EXPA11, EXPA14, EXPB3, and EXPB1-like), six genes encoding xyloglucan endotransglucosylase/hydrolase (XTH5, XTH7, XTH9, XTH22, XTH25, and XTH28), and five members of the glycosyl hydrolase family 17 (BG2, BG3, BG6, BG10, and BG11), which can loosen wall-like networks and control multiple types of growth and development in plants (Cosgrove, 2005). Most of them showed higher expression levels at the early stage of bulbil growth (T1 and T2) (Fig. 4B). Importantly, EXPANSINS can control the formation of the AM (Fleming *et al.*, 1997; Zenoni *et al.*, 2011). We found that three EXPANSINS (EXPA11, EXPA14, and EXPB3) showed peak abundance at T1 and subsequently were reduced 2.3-, 2.2-, and 4.8-fold at T2, respectively, suggesting a localized drive for initiating the hump-like BP (Fig. 1C).

### Transcriptional profiles of hormone candidates: auxin, CK, and ABA

**Fig. 5.**
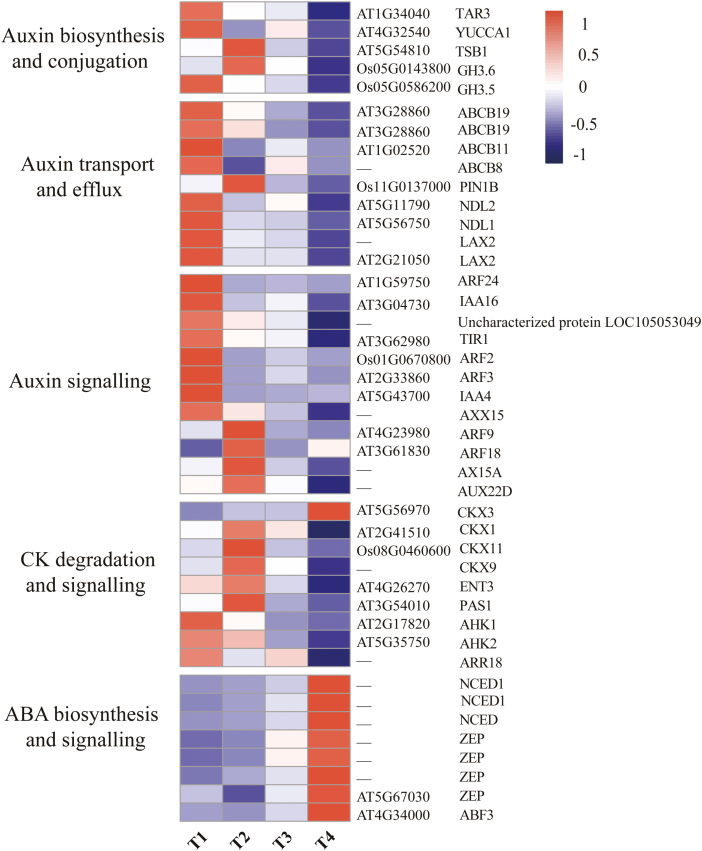
Heat maps of gene sets associated with hormone (auxin, CK, and ABA) biosynthsis and catabolism, transport, and signaling. The expression levels are standardized per gene into *Z*-scores and colored in red and blue for high and low expression, respectively. Gene names (shown on the right) are described in detail in Supplementary Dataset S1.

To investigate hormonal regulation during bulbil growth, we analyzed expression changes of genes involved in auxin, CK, and ABA biosynthesis, transport, and signaling (Supplementary Dataset S1). For auxin, three genes, *TRYPTOPHAN AMINOTRANSFERASE RELATED3* (TAR3), YUCCA1, and tryptophan synthase (TSB1), encoding intermediate enzymes involved in auxin biosynthesis, were detected. Among them, TAR3 and YUCCA1were T1-specifically expressed and showed peak abundance at the T1 stage; TSB1 was identified as a T2-specifically expressed gene, showing the highest expression at this stage. In addition, two *INDOLE-3-ACETIC ACID-AMIDO SYNTHETASES* (GH3.5 and GH3.6) involved in auxin conjugation (Chen *et al.*, 2010), were identified as T1- and T2-specifically expressed, and exhibited peak expression at the T1 and T2 stages, respectively (Fig. 5).

We also detected nine genes encoding proteins involved in auxin transport and efflux, namely transmembrane-targeted efflux carrier PIN1B, members of the ABC transporter B family (ABCB8/11 and two homologs of ABCB19), two homologs of auxin transporter-like protein 2 (LAX2), and NDL proteins (NDL1and NDL2). As expected, PIN1B displayed peak abundance at T2 as a T2-specific gene, and the remaining genes were specifically expressed at T1 and had the highest expression levels at this stage (Fig. 5). In particular, NDL proteins (NDL1 and NDL2) acting as positive regulators in the signaling pathway that modulate auxin transport (Mudgil *et al.*, 2009), were highly expressed at the bulbil initiation stage (T1) and reduced ~2-fold at T2. Interestingly, key components of auxin signaling include AUX/IAA co-receptors (Aux/IAAs) and (ARFs) (Guilfoyle and Hagen, 2007), which had similar expression trends with obvious evidence of stage specificity (Fig. 5). Among them, multiple IAA and ARF genes (IAA4, IAA16, ARF2, ARF3, Axx15, and AX15A-like) were grouped into T1-spcific genes, which have also been implicated in other vegetative developmental growth (Faivre-Rampant *et al.*, 2004; Deng *et al.*, 2012; Jung *et al.*, 2015), and another four genes (ARF9, ARF18, AX15A, and AUX22D) were grouped as T2-specific genes with peak expression levels at T2.

CKs can promote axillary bud outgrowth (Ferguson and Beveridge, 2009). We found that four CK dehydrogenases (CKXs) that decrease CK accumulation (Frébortová *et al.*, 2010) had lower expression levels at T1 relative to other stages (Supplementary Dataset S1). The four genes appeared to be obviously stage specific (namely T2, CKX1, CKX9, and CKX11; and T4, CKX3), showing the highest expression at T2 or T4 (Fig. 5). Remarkably, no CK biosynthetic genes were detected. Although not an SS gene, ENT3 that participates in CK transport (Hirose *et al.*, 2005) was highly expressed (339 FPKM) at T2 but then was strongly reduced 5.6-fold at T4. PAS1 (AT3G54010) that controls the cell division and differentiation mediated by CK (Harrar *et al.*, 2003) was highly induced at T1 and T2, with peak expression at T2. Two histidine kinases (AHK1/2) as CK receptors and a CK-inducible response regulator (ARR18) were observed, and had the highest expression levels at T1.

Interestingly, all SS genes involved in ABA synthesis and signaling were identified as T4-specifically expressed and had the highest expression levels at T4 (Fig. 5; Supplementary Dataset S1). For instance, three genes encoding the rate-limiting ABA biosynthetic enzyme 9-*cis*-epoxycarotenoid dioxygenase (NECD) were expressed with an average 17-fold increase from T1 to T4. Similarly, the average expression level of four zeaxanthin epoxidases (ZEPs) responsible for regulating intermediates in ABA biosynthesis was elevated 2.5-fold at T4 relative to T1. In addition, an ABA-INSENSITIVE protein (ABF3) binding to the ABA-responsive element (Kim *et al.*, 2004) was up-regulated 3-fold at T4 relative to T1. The expression changes for ABA-related genes provided obvious evidence that enhanced ABA biosynthesis and signaling exist at the later stage of bulbil growth (T4).

Further, to validate the involvement of auxin, CKs, and ABA during bulbil growth, we tested their contents and analyzed their correlation with the expression of hormone-related genes (Table 1; Supplementary Fig. S6). The highest amount (37.54 ng g^−^1) of IAA at T1 was observed and showed a high correlation with increased expression of auxin biosynthetic genes (TAR3 and YUCCA1) (*R*^2^ >0.83), demonstrating the localized production of auxin in bulbils. GH3.5 and several of the transport proteins mentioned above (ABCB19, LAX2, and NDL1) were also tightly linked to auxin levels (Supplementary Fig. S6), which provides a mechanism for coping with excess auxin by conjugation and transportation. The high contents of CKs (zeatin and dihydrozeatin) existing at T1 and T2 (Table 1) were coincident with the repression of CKX1 and CKX9, and the activation of ENT3. The ABA content was lowest at T1 and T2, but rose sharply at subsequent stages, which coincides with expression profiles of NCEDs and ZEPs (*R*^2^ >0.89) (Supplementary Fig. S6).

**Table 1.** Levels of phytohormones (IAA, CK, and ABA) in 1g of bulbil tissue

Time point	IAA	CK		Total CKs	ABA
		Zeatin	Dihydrozeatin		
T1	37.54±2.25***	101.92±8.49	26.05±1.58	127.97±8.66	1.47±0.21
T2	10.90±1.20	122.90±3.44**	29.71±1.77*	152.61±4.34**	4.54±0.74
T3	1.70±0.58	56.35±3.72	19.03±0.99	75.38±4.43	20.72±2.45
T4	0.49±0.45	46.14±3.07	6.00±1.75	52.14±2.10	32.82±3.69**

Values are shown in ng g^–1^ (means ±SD). Asterisks represent statistical differences between stages (*P<0.05; **P<0.01;***P<0.001).

### Starch and sucrose metabolic processes

**Fig. 6.**
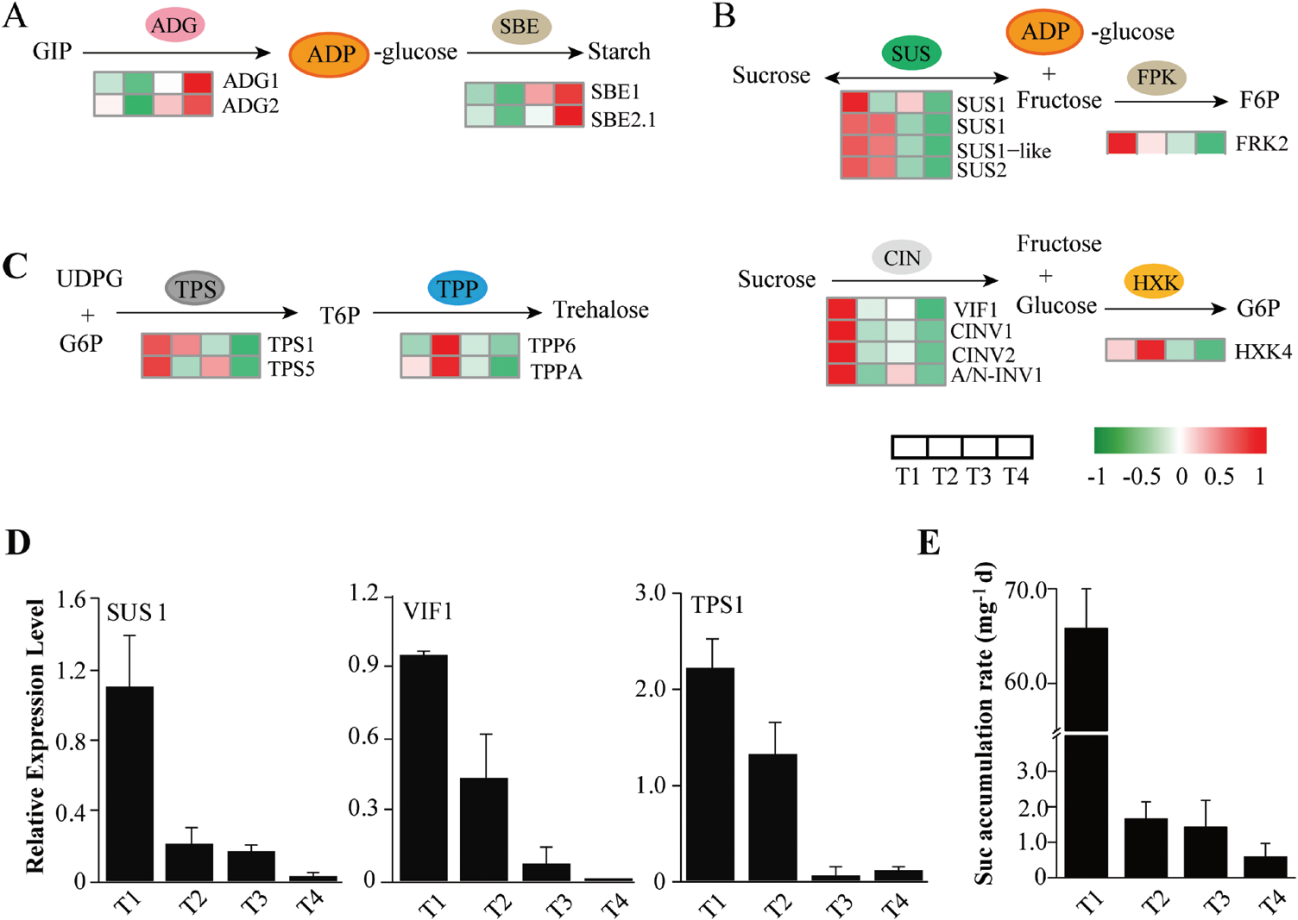
Gene and metabolite regulation involved in starch and sucrose metabolic processes, and sucrose signaling. (A) Starch biosynthesis process. (B) Sucrose metabolism. (C) Sucrose signaling involved in the trehalose metabolism pathway. Heat maps next to the arrows represent changes in expression of genes encoding corresponding enzymes for reactions. The expression levels are standardized per gene into *Z*-scores and colored in red and green for high and low expression, respectively. (D) Verification of expression levels of genes encoding SUS1, VIF1, and TPS1 determined by qRT–PCR. (E) Sucrose accumulation in bulbils, demonstrating that sucrose is sharply increased at the early stage of bulbil formation (T1). Data show the mean ±SD (*n*=5). ADG, glucose-1-phosphate adenylyltransferase; CIN, cell wall invertase; FRK, fructokinase; HXK, hexokinase; SBE, glucan-branching enzyme; SUS, sucrose synthase; TPP, trehalose-phosphate phosphatase; TPS, trehalose-phosphate synthase; VIF1, vacuolar invertase1.

We predicted several marker genes involved in starch synthesis (Supplementary Dataset S1), such as genes encoding the small subunit of Glu-1-P adenylyltransferase (ADG1/2), and glucan-branching enzymes (SBE1 and SBE2.1), which consistently presented the highest expression levels at T4 (Fig. 6A). Two genes (ADG2 and SBE2.1) were T4-specifically expressed according to the SS method. This result was consistent with that of GO-enriched analysis, demonstrating an enriched wax biosynthetic process at T4 (Fig. 3B). In addition, there were multiple genes encoding sucrose synthases (SUSs) and invertases, representing key genes that participate in sucrose metabolism (Fig. 6B; Supplementary Dataset S1). Four genes encoding SUSs (three homologs of SUS1 plus SUS2-like) that can reversibly catalyze sucrose biosynthesis were significantly up-regulated at T1 and gradually decreased at the next stages. In particular, SUS1 (c130514.graph_c0) showed a highly expressed level with an FPKM value >2000. Meanwhile, we found that four T1-specific SS genes encoding invertases (VIF1, CINV1, CINV2, and A/N-INV1) peaked at T1, which can degrade sucrose and produce free hexoses. More importantly, we observed several members of the trehalose-phosphate synthase (TPS) and trehalose-phosphate phosphatase (TPP) families which have been implicated in the regulation of axillary bud development through sugar signaling (Fichtner *et al.*, 2017). Among them, two TPS genes (TPS1 and TPS6) peaked at T1 (Fig. 6C); these can produce trehalose-6-phophate (T6P) as an important sucrose signal affecting plant development (O’Hara *et al.*, 2013; Figueroa and Lunn, 2016). In contrast, two TPP genes (TPP6 and TPPA) with the role of dephosphorylating T6P showed lower expression levels at T1 and peaked at T2. The result of this appeared to be an enhanced T6P pathway in bulbils at the early stage (T1).

Given the crucial role in triggering axillary bud outgrowth by sucrose (Barbier *et al.*, 2015), we further detected the expression levels of some key genes involved in sucrose metabolism by qRT–PCR technology, and detected sucrose accumulation in bulbils at different stages. Three representative genes SUS1, VIF1, and TPS1 involved in this process showed peak expression profiles at T1 (Fig. 6D), which was consistent with the results of transcriptome analyses. Importantly, we observed that the level of sucrose was sharply elevated with an accumulation rate of 65.8 mg d^−1^ during bulbil initiation and increased slowly in the subsequent stages (Fig. 6E).

### Transcription regulators (TFs) involved in yam bulbil growth

**Fig. 7.**
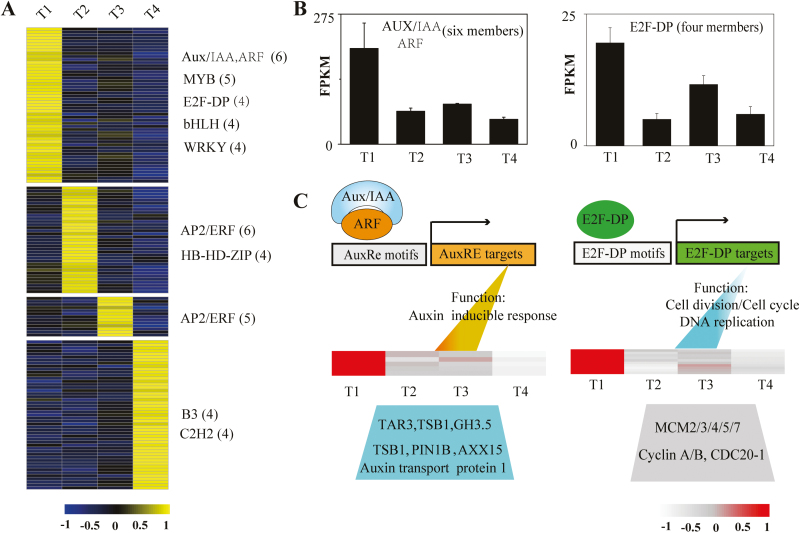
Expression profiles of transcription factors (TFs) and hypothetical regulatory modules. (A) Heat map of scaled FPKM values of 149 stage-specific TFs (with SS score >0.3) identified during bulbil growth. The TF families listed on the right show predominantly expression in corresponding stage-specific clusters. The numbers in parentheses represent the number of members from TF families in this cluster. (B) Cumulative expression profiles of AUX/IAA (six members) and E2F-DP (four members) TFs in the T1-specific cluster. (C) Hypothetical models of AUX/IAA- and E2F-DP- mediated gene regulation in the T1-specific cluster. The heat map shows the expression of genes involved in GO functional categories by AUX/IAA and E2F-DP induction, suggesting that the two TFs and their targets are co-over-represented in the T1-specific cluster.

We searched the SS gene set for the over-representation of families of TFs from The Arabidopsis Information Resource (TAIR) (Perez-Rodriguez *et al*., 2010), and predicted 149 TFs as stage-specifically expressed. Most of them are members of the AUX/IAA, AP2/ERF, WRKY, bHLH, MYB, C2H2, and C3H families (Supplementary Dataset S1). Consistent with the proportion of all SS genes captured during bulbil developmental stages (Fig. 3A), T1 and T4 type bulbils had greater numbers of stage-specific TFs relative to the other two types, with 52 and 49 TFs, respectively. In contrast, the T3 type had the lowest number of TFs (13), and 35 TFs were identified as T2-specifically expressed. The expression profiles of these stage-specific TFs are shown in Fig. 7A.

Notably, we here focused on T1-specifically expressed TFs due to their possible roles in regulating the initiation of the bulbil. Strikingly, six members of the Aux/IAA and ABF protein families showed 3-fold accumulation induction at T1 relative to the next stage (T2) (Fig. 7B), and are required for regulating auxin response genes in plant developmental processes including AM initiation (Deng *et al.*, 2012; Jung *et al.*, 2015; Kato *et al.*, 2017). Generally, TFs and their targets are co-expressed in the same gene cluster. We found that some genes involved in auxin metabolism, signaling, and transport, including GH3.5, TAR3, PIN1B, AXX15, and LAX2, were grouped into the same cluster and over-represented at T1 (Fig. 7C). Most of them are potentially targeted by Aux/IAAs and ARFs (Guilfoyle and Hagen, 2007). In addition, the E2F-DP family are important regulators of the cell cycle and directly target various genes related to cell division and DNA replication (De Veylde *et al*., 2002). Four E2F-DP TFs exhibited a 6.8-fold induction of accumulation at T1 compared with T2 (Fig. 7B). Genes including multiple CYCA/B genes and DNA replication licensing factors (MCM2/3/4/5/7) associated with the cell cycle and DNA replication were also enriched at T1 and peaked in expression levels, which probably resulted from the over-representation of E2F-DP TFs at T1 (Fig. 7C).

In addition to AUX/IAA and E2F-DP families, MYB, WRKY, and bHLH were other TF families over-represented in the T1 cluster and had the highest expression at T1. For instance, WRKY TFs have been implicated in the regulation of AMs (Guo *et al.*, 2015); the accumulated expression of four WRKY TFs at T1 was 1.6-fold higher relative to other stages. Some MYB TFs affect the pattern of lateral meristem initiation (Hsu *et al.*, 2015), five MYB TFs represented a functional category at T1, all of which had 2-fold higher expression at T1 relative to other stages. Similarly, four bHLH TFs were highly induced, with 2- to 11-fold higher expression at T1. To further confirm the regulatory role of TFs, we selected three TFs, MYB (C126446.graph-c0), WRKY (C125026.graph_c1), and NAC (C116834.graph_c0), due to their higher expression levels and key roles in promoting AM initiation in other plants (Yang and Jiao, 2016), and examined their expression at the early initiation of bulbil formation by *in situ* hybridization (Supplementary Fig. S8). Consistent with the RNA-seq results, we found that all of them were specifically expressed in the AM initiation zone (a dome-shaped tissue) at the T1 stage, and strongly decreased with the thin chromogenic signals at the next developmental stages, indicating the key roles that they play during bulbil initiation.

## Discussion

### Identification of gene sets associated with yam bulbil growth

In this study, we performed comprehensive transcriptome profiling of yam bulbil, demonstrating a dramatic shift in gene expression for different bulbil types. In particular, we used the SS scoring algorithm to obtain stage-specifically expressed gene sets that highlight the nature of different bulbil types. These gene sets were enriched in some known biological processes and KEGG pathways associated with gene regulation of axillary bud growth, providing a good resource for identifying key candidates controlling bulbil growth. As shown in Fig. 4A, the T1-specific cluster identified a set of genes encoding cell cyclin proteins (CYCA1-1, CYCA3-2, CYCA3-4, CYCB2-1, and CYCB3-1), kinesins (KIN5C, KIN4A, KIN10A, and NACK1), and DNA replication licensing factors (MCM2/3/4/5/7) involved in the cell cycle and proliferation (Supplementary Dataset S1). In the control of axillary buds, cell cycle-related genes perform a quality control function in promoting the resumption of meristem organogenic activity. This fact is directly supported by a variety of experiments showing that increased expression of cell cycle genes in dormant buds from pea and Arabidopsis by decapitation stimulates bud outgrowth (Devitt and Stafstrom, 1995; Shimizu and Mori, 1998). Similar evidence has shown that decreased expression of cell cycle genes (CYCB, CYCD2, and CDKB) represses bud growth in *Sorghum bicolor* (Kebrom *et al.*, 2010). In apple plant, the expression of cell proliferation genes (kinesins) is up-regulated at the early stage of axillary bud formation (Tan *et al.*, 2019). Consistent with these findings, a large set of cell cycle/division genes were T1-specifically expressed and showed peak abundance at this stage (Fig. 4A), which were probably regulated by E2F-DP TFs over-represented at this stage (Fig. 7C). This gene expression profile increases the vitality of cell division in leaf primordia meristems and allows the resumption of organogenesis of young bulbils.

Several lines of evidence suggest that the expression of genes associated with cell wall expansion, such as EXPANSINS, determines the rate of the initiation and elongation of premature AMs, and exerts a profound influence on plant development and morphology (Fleming *et al.*, 1997). In a reported experiment from *Petunia hybrida*, overexpression of the PHEXPA1 gene promotes AM release, whereas silencing PHEXPA1 produces opposite phenotypes (Zenoni *et al.*, 2011). The enhanced expression of RhEXP by a CK signal activates the shoot apical meristem (SAM) organogenic activity in rose axillary buds and further promotes bud elongation (Roman *et al.*, 2016). In addition to EXPANSINS, glycosyl hydrolase family 16/17 (XTHs/BGs) involved in cell wall modification have been implicated in the regulation of cell elongation during development of various plant organs (Cosgrove, 2005). Consistent with these observations, we detected 15 gene sets of EXPANSINS from EXPA/B and GH 16/17 families, and found a distinctly up-regulated expression at the early stage of bulbil formation (T1) (Fig. 4B; Supplementary Dataset S1). Most of them were defined as T1-specifically expressed. Such results may be explained by the fact that the set of EXPANSINS, XTH, and BG genes contributes a unique role in maintaining cell enlargement during bulbil initiation and building the special architectural form.

Starch constitutes most of the biomass of the mature bulbil, accounting for 50–80% of its dry matter, and is a main trait being improved by breeding (Tamiru *et al.*, 2008). As expected, we observed multiple marker genes (ADG1/2 and SBE1/2.1) involved in the starch biosynthesis pathway (Fig. 6A). Of particular interest are the SBE genes that control amylopectin production, which showed relatively high expression levels at the later stages of bulbil growth (T4). These results are consistent with previous observations in maize embryo and endosperm development (Chen *et al.*, 2014). The highly expressed SBE genes may be used to improve the quality of the bulbil by further genetic manipulation.

### Bulbil growth requires the coordinated control of hormone-related genes

Plant hormones and their interactions have long been considered to play central regulatory roles in controlling axillary bud growth (Domagalska and Leyser, 2011; Yang and Jiao, 2016). Here, T1-specifically expressed TAR3 and YUCCA1 genes involved in auxin biosynthesis were highly up-regulated at T1 (Fig. 5); these genes were directly responsible for the localized production of auxin in bulbils at the early stage (Table1). Consistent with our observation, localized auxin biosynthesis is required for AM initiation in maize and Arabidopsis (Gallavotti *et al.*, 2008; Zhao, 2010). Knockout of YUCCA genes leads to fewer branches due to the absence of AMs (Cheng *et al.*, 2006). A similar result had been found in that auxin promotes the initiation of upper bulbils in *L. lancifolium*, but inhibits their further growth (Yang *et al.*, 2017). On the other hand, the ATC model supports that auxin needs to be exported from axillary buds for its outgrowth by establishing the localized PAT stream in the bud stem (not in the main stem) (Blilou *et al.*, 2005). The PAT stream is driven by PIN proteins, auxin transporter-like protein 2 (LAX2), and members of the ABC transporter B family belonging to auxin efflux carriers that can facilitate auxin export out of cells (Petrášek and Friml, 2009). Arabidopsis mutants with more axillary growth increase PIN protein levels and the amount of auxin moving by the PAT stream (Bennett *et al.*, 2006). In pea plants, increased auxin export from buds is accompanied by PIN1 polarization after decapitation, and further activates bud outgrowth (Balla *et al.*, 2011). In accordance with this evidence, we observed the over-representation of auxin efflux proteins, including ABCB-type transporters (ABCB8/11/19), LAX2, and NDL1/2 that were closely correlated with the auxin level (Supplementary Fig. S6), whereby these transport proteins facilitate transport of excess auxin and trigger bulbil growth (Table1).

Outgrowth of axillary buds is positively correlated with CK levels that are inhibited by the mobile auxin in the main stem (Ferguson and Beveridge, 2009; Domagalska and Leyser, 2011). The CK levels in chickpea buds increase 25-fold after decapitation, suggesting that CKs are necessary to initiate bud growth (Turnbull *et al.*, 1997). In some cases, CKs can stimulate bud outgrowth by direct application to buds, even in the presence of apical auxin (Dun *et al.*, 2012). In *phyB* sorghum mutants, reduced expression of genes involved in CK biosynthesis and signaling leads to resistance to bud outgrowth (Kebrom and Mullet, 2016). Mutations in rice CKX that degrade CK give rise to increased panicle branches and spikelet numbers in the inflorescence (Ashikari *et al.*, 2005). Here, we did not find any CK synthetic genes, but observed the repression of degradation genes (CKX1, CKX3, CKX9, and CKX11) in bulbils at the T1 stage (Supplementary Dataset S1), indicating that the presence of CK (Table 1) in the bulbil probably stemmed from the adjacent stem by CK flow. This speculation was strongly supported by the fact that the highly expressed CK transporter (ENT3) is tightly linked to CK levels (*R*^2^ >0.95) (Supplementary Fig. S6). In addition, a CK-inducible response regulator (ARR18) and two CK receptors (AHK1/2) were identified as T1-specifically expressed, suggesting an enhanced CK signaling at the T1 stage. Taken as a whole, the expression of the CK-related genes above could drive the promotion of bulbil growth at the initiation stage.

ABA has been thought to be a key component of regulating axillary organ growth. For a variety of plant species, the decline of ABA levels after decapitation precedes the onset of bud outgrowth (Zheng *et al.*, 2015). In Arabidopsis, elevated ABA delays bud outgrowth and decreases elongating buds under low red:far-red (R:FR) conditions (Reddy *et al.*, 2013; Yao and Finlayson, 2015). Several hypotheses have been postulated suggesting that ABA acts downstream of auxin and stringonolactone, possibly as a second messenger (Cline and Oh, 2006; López-Ráez *et al.*, 2010). Independently of this presumption, it is undoubted that increased expression of genes involved in ABA biosynthesis is linked to repression of axillary bud growth. Consistent with previous reports, our data revealed that three NECD genes and four ZEP genes responsible for regulating ABA synthesis were down-regulated at T1 and then strongly up-regulated at T4 (as T4-specifically expressed genes) (Fig. 5). In particular, the ABA level was closely linked to the expression of NECDs and ZEPs (Supplementary Fig. S6). Thus, these genes could decrease ABA accumulation in bulbils at the initiation stage (Table 1), thereby promoting bulbil growth.

On the other hand, we attempted to establish a linkage of phytohormone signals and functional gene sets associated with bulbil growth. We found that levels of multiple phytohormones (auxin, CKs, and ABA) were tightly correlated with transcriptional expression of most identified genes involved in the functional categories cell division, proliferation, and expansion (Supplementary Fig. S7). This close linkage was also found in other investigations on axillary bud development (Reddy *et al.*, 2013; Roman *et al.*, 2016; Majda and Robert, 2018), which provides evidence that mutiple phytohormones are likely to act as integrated signals that provide a forward force to genes related to cell division, proliferation, and expansion, thus promoting bulbil growth.

### The key mechanism of bulbli initiation is modulated through sucrose supply and signaling

In addition to phytohormone signals, sugar (sucrose or its analogs), as a novel player, contributes to the activation of axillary bud growth (Barbier *et al.*, 2015). In diverse plant species after decapitation, the progressive decrease of auxin levels in the stem is too slow to dominate the early bud formation, whereas sugars are rapidly redistributed and enter buds to promote their growth (Barbier *et al.*, 2015). From a representative experiment in pea plants, the rate of PAT is 1 cm h^−1^ when apical dominance is lost, yet the outgrowth of the bud reaches 40 cm at 2.5 h after decapitation (Mason *et al.*, 2014). In *Narcissus* plants, direct application of sucrose greatly increases the percentage of bulbil production (Staikidou *et al.*, 1994). In our study, the rapid rate of accumulationof sucrose was observed at T1 (Fig. 6E), which facilitates triggering the release of bulbils. Moreover, the sucrose accumulation level had positive effects on transcriptional expression of gene sets associated with cell division, and expansion (Supplementary Fig. S7). Transcriptome analysis revealed that the expression of key genes (SUSs and CINs) involved in sucrose metabolism was highly up-regulated at T1 (Fig. 6B), which can unblock the process of sucrose supply in a timely manner. More importantly, increased CIN expression can positively regulate axillary bud initiation by generating sugars for trophic uptake under the interplay of light and phytohormones (Rabot *et al.*, 2014).

On the other hand, sucrose functions as a critical signal through regulating the pathways involving T6P and HXK1 (O’Hara *et al.*, 2013). HXK1-overexpressing Arabidopsis lines show enhanced branching (Kelly *et al.*, 2012). In particular, the elevated T6P level has been implicated to be the signal that increases sugar influx into buds after decapitation, whereby buds are released from dormancy and elongated (Yadav *et al.*, 2014; Figueroa and Lunn, 2016). T6P is synthesized by TPS and dephosphorylated to TPP by TPP genes. Overexpression of *Escherichia coli* TPS (OtsA) in Arabidopsis results in a rise in T6P levels and triggers the proliferation of shoot branching; on the other hand, overexpression of *E. coli* TPP (OtsB) decreases both the T6P level and shoot branching (Schluepmann *et al.*, 2003). Similar evidence demonstrated that Arabidopsis lines constitutively affected in synthesis and degradation of T6P increase and reduce branching phenotypes, respectively (Yadav *et al.*, 2014). In garden pea, T6P was found to be the signal of sucrose availability to promote outgrowth of axillary buds (Fichtner *et al.*, 2017). In our study, we observed the induction of TPS genes (TPS1 and TPS5) and the inhibition of TPP genes (TPP6 and TPPA) at the initiation stage of the bulbil (T1) (Fig. 6C), which is more likely to be a consequence of enhanced sucrose signaling. These up-regulated TPS genes could increase T6P levels, thereby contributing to the promotion of bulbil growth.

### Requirement for transcription regulators for bulbil initiation

The initiation of AMs is tightly linked to the activity of bud-specific regulators that can regulate transcription responses of functional genes through binding motifs in their promoters. Most of the regulators are members of multiple TF families, such as known R2R3 MYB proteins (*REGULATOR OF AXILLARY MERISTERMS*, *RAX*) from Arabidopsis, NAC domain TFs (*CUP-SHAPED COTYLEDON*, *CUC*), WRKY domain protein (*EXB1*), GRAS domain protein (*LATERAL SUPPRESSOR*, *LS*) in tomato, and TCP TFs (*BRANCHED1/2*) in Arabidopsis and in sorghum (*TB1*) (Janssen *et al.*, 2014; Yang and Jiao, 2016). Genetic studies of mutant plants have demonstrated that these transcriptional activators are specifically expressed in the boundary zone between the leaf primordium and the SAM, and control the fate of AM initiation and the production of buds (Keller *et al.*, 2006; Yang *et al.*, 2012; Guo *et al.*, 2015). For instance, loss of the *RAX1* gene encoding MYB37 in Arabidopsis or its orthologous gene *BLIND* (*BL*) in tomato leads to failure to generate lateral buds during vegetative development (Keller *et al.*, 2006; Naz *et al.*, 2013). Overexpression of *EXB1* encoding WRKY71 increases excessive AM initiation and bud activities, and produces bushy and dwarf phenotypes by transcriptionally regulating *RAX* genes (Guo *et al.*, 2015). In this study, we verified a large set of TF genes during bulbil developmental stages, especially the over-represented TFs from Aux/IAA, E2F-DP, MYB,WRKY, and bHLH families that are T1-specifically expressed with the highest expression at this stage (Fig. 7A). In the same cluster, these TFs directly resulted in the over-representation of their targeted genes by putative regulatory modules. In the T1 cluster, two regulatory modules of Aux/IAA and E2F-DP were speculated to exist, and caused the over-representation of genes linked to auxin-inducible responses, cell division, and DNA replication (Fig. 7C), respectively. Three T1-specifically expressed TFs from WRKY, NAC, and MYB were confirmed to be highly expressed in the meristematic cell zone at the early stage of bulbil formation (Supplementary Fig. S8). These data suggested that transcriptional regulators are required for the early step of bulbil expansion. However, regulatory modules, and the downstream genes targeted by them, still need to be explored by genetic interaction experiments in future studies.

### Conclusion

**Fig. 8.**
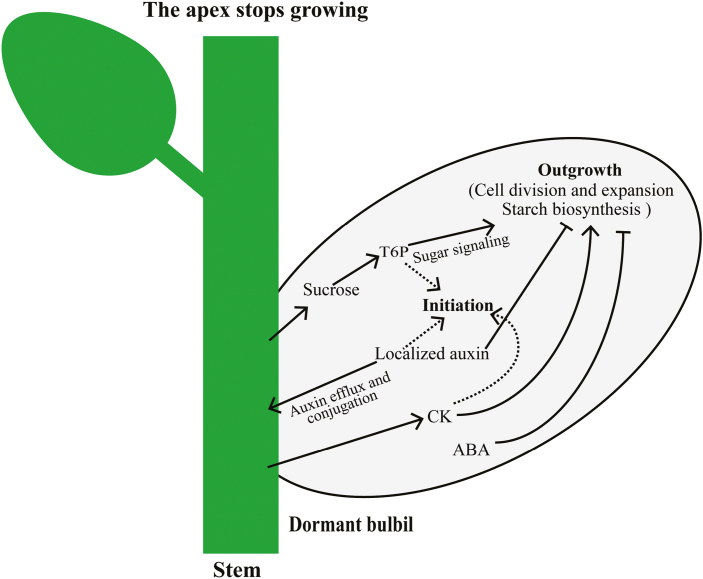
Schematic model of gene regulation by auxin, CK, ABA, and sucrose during bulbil growth. The localized auxin in the bulbil is transiently produced by stimulation of auxin synthesis (TAR3 and YUCCA1), and activates bulbil initiation. Further, the excess auxin is depleted and transported to maintain bulbil outgrowth by up-regulation of auxin-conjugated genes (GH3.5 and GH3.6) and auxin efflux proteins (PIN1B, LAX2, and the ABC transporter B family). CKs promote its growth through rapid activation of the CK transporter (ENT3) gene, together with suppression of CK degradative genes (CKX1/3/9/11). ABA contributes to bulbil growth through repression of ABA synthesis genes (NECDs and ZEPs) at the early stage of bulbil formation. Sucrose probably acts as a signal, and promotes bulbil growth through up-regulation of genes involved in the T6P pathway (TPS1 and TPS5). The integration of these hormones and sucrose provides a forward signaling to activate genes associated with cell division, proliferation, and expansion, which contributes key roles in maintaining cell enlargement during bulbil growth.

Taken together, bulbil transcriptomic data, especially for stage-specifically expressed gene sets, provide a valuable new resource that can be queried to obtain important candidates regulating yam bulbil initiation and growth. A schematic model based on our results is proposed in Fig. 8. Compared with previous reports discussed above, more direct evidence was presented here to reveal the key regulatory programs of bulbil formation. We found that metabolite levels of multiple phytohormones (auxin, CKs, and ABA) and sucrose are highly coincident with transcription changes of genes involved in cell division, proliferation, and expansion. Furthermore, the integration of auxin, CKs, ABA, and sucrose provides a forward signaling to these functional genes that contribute unique roles in maintaining cell enlargement and lead to bulbil initiation and growth. In particular, the localized production of auxin in bulbils is transiently required to trigger bulbil formation, but can be exported to maintain bulbil outgrowth by up-regulating various auxin efflux proteins (PIN1B, LAX2, and the ABC transporter B family). In addition, we have identified several T1-specifically expressed TFs from Aux/IAA, E2F, MYB, WRKY, and bHLH families, and described their key role in triggering bulbil formation by the prediction of regulatory modules or *in situ* hybridization experiments. Such work as presented here allows, to our knowledge for the first time, an increased understanding of the complex gene regulatory framework underlying yam bulbil growth. Further work will contribute to identification of the role of candidate genes in regulating bulbil growth by misregulation and genetic transformation.

## Supplementary data

Supplementary data are available at *JXB* online.

Table S1. List of the primer sequences used for qRT–PCR analyses.

Table S2. Summary of RNA-seq reads in the yam bulbil transcriptome.

Table S3. Assembly statistics for the yam bulbil transcriptome.

Table S4. Lists of differentially expressed genes.

Table S5. List of the most enriched GO terms for DEGs.

Table S6. Lists of significantly enriched KEGG pathways.

Table S7. Gene set of stage-specifically expressed genes.

Dataset S1. Tables of candidate genes and regulators associated with bulbil growth.

Fig. S1. Pearson correlation relationship between biological replicates.

Fig. S2. Validations of gene expression profiles by qRT–PCR.

Fig. S3. Hierarchical clustering of all DEGs across different stages.

Fig. S4. The most enriched GO terms for all DEGs.

Fig.S5. Enriched KEGG pathways.

Fig. S6. Correlations between hormone-related gene expression and its metabolite levels.

Fig. S7. Correlations between levels of hormones, sucrose, and the expression of genes involved in cell division, proliferation, and expansion.

Fig. S8. RNA *in situ* hybridization for MYB, WRKY, and NAC transcription factors.

erz552_suppl_Supplementary_Dataset_S7

erz552_suppl_Supplementary_Tables_S4-S7

erz552_suppl_Supplementary_Legends

erz552_suppl_Supplementary_Tables_S5-S6

erz552_suppl_Supplementary_Tables_S1-S3

erz552_suppl_Supplementary_Figures_S1-S8
