# Supplementary material for: Morphological and stage-specific transcriptome analyses reveal distinct regulatory programs underlying yam (Dioscorea alata L.) bulbil growth
Source: J Exp Bot. 2019 Dec 13;71(6):1899–914. doi: 10.1093/jxb/erz552 (PMC7242083; doi:10.1093/jxb/erz552)
Supplement: erz552_suppl_Supplementary_Legends [file erz552_suppl_supplementary_legends.docx]

**Supplementary data**

Supplementary data are available at *JXB* online.

**Table S1.** List of the primer sequences used for qRT-PCR analyses.

**Table S2.** Summary of RNA-seq reads in yam bulbil transcriptome.

**Table S3.** Assembly statistics for yam bulbil transcriptome.

**Table S4.** Lists of differentially expressed genes.

**Table S5.** List of the most enriched GO terms for DEGs.

**Table S 6.** Lists of significantly enriched KEGG pathways.

**Table S7.** Gene set of stage-specific expressed.

**Dataset S1.** Tables of candidate genes and regulators associated with bulbil growth.

**Figure S1.** Pearson correlation relationship between biological replicates.

**Figure S2.** Validations of gene expression profiles by qRT-PCR.

**Figure S3.** Hierarchical clustering of all DEGs across different stages.

**Figure S4**. The most enriched GO terms for all DEGs.

**Figure S5.** Enriched KEGG pathways.

**Figure S6.** Correlations between hormone-related genes expressions and its metabolite levels.

**Figure S7.** Correlations between levels of hormones, sucrose and the expressions of genes involved in cell division, proliferation and expansion.

**Figure S8**. RNA in situ hybridization for MYB, WRKY and NAC transcription factors.
