# Supplementary material for: Morphological and stage-specific transcriptome analyses reveal distinct regulatory programs underlying yam (Dioscorea alata L.) bulbil growth
Source: J Exp Bot. 2019 Dec 13;71(6):1899–914. doi: 10.1093/jxb/erz552 (PMC7242083; doi:10.1093/jxb/erz552)
Supplement: erz552_suppl_Supplementary_Tables_S5-S6 [file erz552_suppl_supplementary_tables_s5-s6.pdf]

Table S5 List of the most enriched GO terms

GO. ID	GO terms	Number of DEGs	P-value	Corrected_P-value
**Biological Process (GO:0008150**				
GO:0006306	DNA methylation	31	6.52E-18	8.32E-15
GO:0051567	histone H3-K9 methylation	27	2.60E-14	3.31E-11
GO:0051726	regulation of cell cycle	22	1.53E-13	1.96E-10
GO:0008283	cell proliferation	26	2.20E-13	2.81E-10
GO:0006275	regulation of DNA replication	22	6.86E-13	8.76E-10
GO:0006261	DNA-dependent DNA replication	18	9.30E-11	1.19E-07
GO:0000911	cytokinesis by cell plate formation	23	2.28E-10	2.92E-07
GO:0080167	response to karrikin	22	5.45E-09	6.95E-06
GO:0016458	gene silencing	10	1.90E-08	2.43E-05
GO:0009611	response to wounding	27	8.84E-08	0.000112827
GO:0031047	gene silencing by RNA	15	8.94E-08	0.000114121
GO:0019288	isopentenyl diphosphate biosynthetic process, methylerythritol 4-phosphate pathway	34	1.94E-07	0.000248318
GO:0019252	starch biosynthetic process	27	2.37E-07	0.000303031
GO:0010075	regulation of meristem growth	16	2.46E-07	0.00030454
GO:0031048	chromatin silencing by small RNA	13	2.53E-07	0.000322463
GO:0006468	protein phosphorylation	112	7.29E-07	0.000930987
GO:0016444	somatic cell DNA recombination	9	1.12E-06	0.001436329
GO:0009409	response to cold	41	1.46E-06	0.001864062
GO:0006342	chromatin silencing	14	1.69E-06	0.002160777
GO:0009813	flavonoid biosynthetic process	11	2.17E-06	0.002769149
GO:0009416	response to light stimulus	21	2.33E-06	0.002981311
GO:0007018	microtubule-based movement	18	4.09E-06	0.005224642
GO:0010189	vitamin E biosynthetic process	6	5.96E-06	0.007616388
GO:0048438	floral whorl development	8	7.38E-06	0.009421973
**Molecular Function(GO:0003674)**				
GO:0004672	protein kinase activity	73	0	0
GO:0016210	naringenin-chalcone synthase activity	11	6.68E-08	5.67E-05
GO:0005488	binding	94	1.29E-07	0.000109534
GO:0008094	DNA-dependent ATPase activity	12	4.47E-07	0.000379851
**Cellular Componen(GO:0005575)**				
GO:0016023	cytoplasmic membrane-bounded vesicle	202	0	0
GO:0005886	plasma membrane	225	9.77E-10	2.07E-07
GO:0048046	apoplast	65	1.76E-09	3.74E-07
GO:0009505	plant-type cell wall	33	5.14E-08	1.09E-05
GO:0009535	chloroplast thylakoid membrane	53	1.67E-07	3.55E-05
GO:0009507	chloroplast	141	3.09E-07	6.55E-05

GO:0009536	plastid	112	4.75E-07	0.000100612
GO:0010287	plastoglobule	21	9.77E-07	0.000207124
GO:0016020	membrane	247	1.50E-06	0.000318542
GO:0005618	cell wall	67	2.18E-06	0.000461655
GO:0005874	microtubule	30	1.59E-05	0.003372371
GO:0009579	thylakoid	21	2.52E-05	0.005336687
GO:0009941	chloroplast envelope	68	2.55E-05	0.005413131

Table S 6. Lists of significantly enriched KEGG pathways ($p < 0.01$)

#Kegg_pathway	ko_id	Cluter_frequency	Genome_frequency	P-value	Corrected_P-value
Starch and sucrose metabolism	ko00500	76 out of 1148	306 out of 10655	0	0
Phenylpropanoid biosynthesis	ko00940	64 out of 1148	217 out of 10655	1.04E-13	1.27E-11
Plant hormone signal transduction	ko04075	67 out of 1148	253 out of 10655	4.67E-13	5.70E-11
Flavonoid biosynthesis	ko00941	29 out of 1148	72 out of 10655	7.22E-11	8.81E-09
Circadian rhythm - plant	ko04712	25 out of 1148	70 out of 10655	2.69E-08	3.28E-06
Phenylalanine metabolism	ko00360	42 out of 1148	174 out of 110655	3.30E-07	4.02E-05
Homologous recombination	ko03440	20 out of 1148	57 out of 110655	9.15E-07	0.000111629
DNA replication	ko03030	22 out of 1148	81 out of 10655	3.03E-05	0.003701157
Amino sugar and nucleotide sugar metabolism	ko00520	51 out of1148	278 out of 10655	9.14E-05	0.011153652
Cyanoamino acid metabolism	ko00460	22 out of 1148	87 out of 10655	9.85E-05	0.012021351
Mismatch repair	ko03430	18 out of 1148	66 out of 10655	0.0001482	0.018080172
alpha-Linolenic acid metabolism	ko00592	22 out of 1148	99 out of 10655	0.0007023	0.085678806
Linoleic acid metabolism	ko00591	6 out of 1148	12 out of 10655	0.0008036	0.098040764
Zeatin biosynthesis	ko00908	8 out of 1148	21 out of 10655	0.0009959	0.121496121
Stilbenoid, diarylheptanoid and gingerol biosynthesis	ko00945	9 out of 1148	27 out of 10655	0.001467	0.178974276
Flavone and flavonol biosynthesis	ko00944	8 out of 1148	24 out of 10655	0.0026827	0.327293834
Carotenoid biosynthesis	ko00906	10 out of 1148	35 out of 10655	0.0029883	0.364571825
Taurine and hypotaurine metabolism	ko00430	7 out of 1148	21 out of 10655	0.0049362	0.602214849
Glycosphingolipid biosynthesis - ganglio series	ko00604	4 out of 1148	8 out of 10655	0.006569	0.801423549
Nitrogen metabolism	ko00910	20 out of 1148	104 out of 10655	0.0070531	0.860482787
