# Supplementary material for: Morphological and stage-specific transcriptome analyses reveal distinct regulatory programs underlying yam (Dioscorea alata L.) bulbil growth
Source: J Exp Bot. 2019 Dec 13;71(6):1899–914. doi: 10.1093/jxb/erz552 (PMC7242083; doi:10.1093/jxb/erz552)
Supplement: erz552_suppl_Supplementary_Tables_S1-S3 [file erz552_suppl_supplementary_tables_s1-s3.pdf]

Supplementary Tables:

Table S1. List of the primer sequences used for qRT-PCR analyses

Gene ID	Gene Name	Forward primer (5'-3')	Reverse primer (5'-3')
c131642.graph_c1	SS4	GTTTCCAATTGCCGCCATTG	ATCGATCCGAAGCAGGCTCA
c129481.graph_c0	SBE1	GGCAAGAGGCTATGCACCAA	CACGGAATCGACACAGCCTT
c119393.graph_c0	ISA1	AGTGGCCTCCCAGCAAGAAC	GGATTGCGAGTTTGGTGGTG
c135828.graph_c1	PWD	TGAGGAGTGGAAACGGAGGA	CGTTCCATGTCTCCCGTGAT
c130514.graph_c0	SUS1	CCACACGGTATAGCCCAGGA	CCATGCCGACTTCATCATCA
c129305.graph_c2	VIF1	TGCTCTACACCGGATCCACC	CGCATCCGACTTTGTCCACT
c118684.graph_c0	SUS1-like	TGAATGGCCACATCAGGTGG	CAGGCTGTACAAAGGCGCCT
c129337.graph_c0	TAR3	CCGACGCAGACAGTGGAAAT	GGTAACGTAGCCCATCCGGT
c126329.graph_c0	PIN1B	GCAGTCGAGCTGCGAGAAGA	AAACCGGAGGCGCTAAATGA
c113758.graph_c0	IAA15	GTGCTGCGCGGTAACTTTGT	TTGAGAACGCTGATCCCCAA
c130573.graph_c1	ARF15	AATGCACTCCACCCGGTTGT	CCTTCCCAGGAGCTTGTTGC
c111520.graph_c0	PhyB	TTTCCTGTCCAATGGTTGCA	GCTCGTCGGAGAGGTTTTTG
c130377.graph_c3	Dio A	GCCAACCTTGACGACTGCAT	AATACTCTCGCCCAGCTCGG
c135265.graph_c3	Dio B	TTCACTGTTGCACGAGCAGC	GTGGCTATGGCATGGAGCAG
c129560.graph_c1	CESA7	GCAACACCATGATGGCCAAC	GGTGTACGTCGGAACCGGTT
c134554.graph_c3	CHI	GCCGCACTATGCATCTCCTG	CCTGTCGAGCATGCCTCTGT
c134934.graph_c0	EGase	GATGGCAAACCCTCAATCGC	TAAGGCCTCCGATGTCCTCG
c124858.graph_c0	EXPA1_like	TGGCCATAGTGCAGTGTGGA	TCCAGACCCATTTTCCGTCA
c114615.graph_c0	NAC	AGTGCATTACCTCTGCCGGA	TACCTGGCAATTCCCAAGGA
c118218.graph_c0	C2H2	GACGTTTAGAGCGTTTGCGC	CCGTAGATGCAATCGCAACC
c116834.graph_c0	AP2	CAAAGGTGTCCGAGTGTGGC	GTTTGGCCTTCTTACCCCGA
c129895.graph_c0	C3H	CGGCCAGAGACAGTGCTGAT	GGCACCTTGGAGGAAACACA
c125026.graph_c1	WRKY	TTGTAGAACGGACGGCTTGG	CACATACGAAGGCGAGCACC
c132918.graph_c1	bHLH	GATCCATTGCACCCCTCCTT	TCAGGTAATCCTCCGCACGA
	EF-1a-F	TCAGGCTGACTGTGCTGTCCT	GTGGTGGCGTCCATCTTGTT

Table S2. Summary of RNA-seq reads in yam bulbil transcriptome

Sample	Replicates	No.base in dataset (after QC)	No.clean reads	Mapped Ratio (%)	⩾Q30(%)
T01	1	16,655,479,736	56,017,015	73.59	94.27
	2	14,579,803,638	49,112,048	72.8	94.19
	3	19,681,813,798	66,142,499	72.18	93.99
T02	1	18,307,036,212	61,563,310	73.76	93.82
	2	17,066,897,702	57,421,095	73.57	93.8
	3	18,132,828,188	60,865,352	74.12	93.89
T03	1	18,176,433,892	61,253,397	71.76	93.5
	2	16,828,368,454	56,561,893	71.94	93.24
	3	15,525,725,344	52,251,678	72.85	93.59
T04	1	15,037,966,578	50,516,227	72.66	93.59
	2	18,097,731,650	61,231,289	72.38	93.88
	3	16,247,396,402	54,773,454	72.37	93.76
T1, T2, T3 and T4 mean the initiation , early, middle and mature stages of bulbil formation, respectively.					

Table S3. Assembly statistics for yam bulbil transcriptome

item	Transcripts	Unigenes
No.of 200-300 bp reads	44,604 (22.38%)	400,88(40.92%)
No.of 300-500 bp reads	27,060(13.58%)	17308(17.67%)
No.of 500-1000 bp reads	41,173(20.66%)	18361(18.74%)
No.of 1000-2000 bp reads	44,579(22.37%)	12195(12.45%)
No. of >2000 bp reads	41,853(21.00%)	10003(10.21%)
Total Number	199,270	97,956
Total Length (bp)	250,797,988	79,527,036
N50 Length (bp)	2,173	1,579
Mean Length (bp)	1,258	812
