# Supplementary material for: Morphological and stage-specific transcriptome analyses reveal distinct regulatory programs underlying yam (Dioscorea alata L.) bulbil growth
Source: J Exp Bot. 2019 Dec 13;71(6):1899–914. doi: 10.1093/jxb/erz552 (PMC7242083; doi:10.1093/jxb/erz552)
Supplement: erz552_suppl_Supplementary_Figures_S1-S8 [file erz552_suppl_supplementary_figures_s1-s8.pdf]

Supplementary Figures:

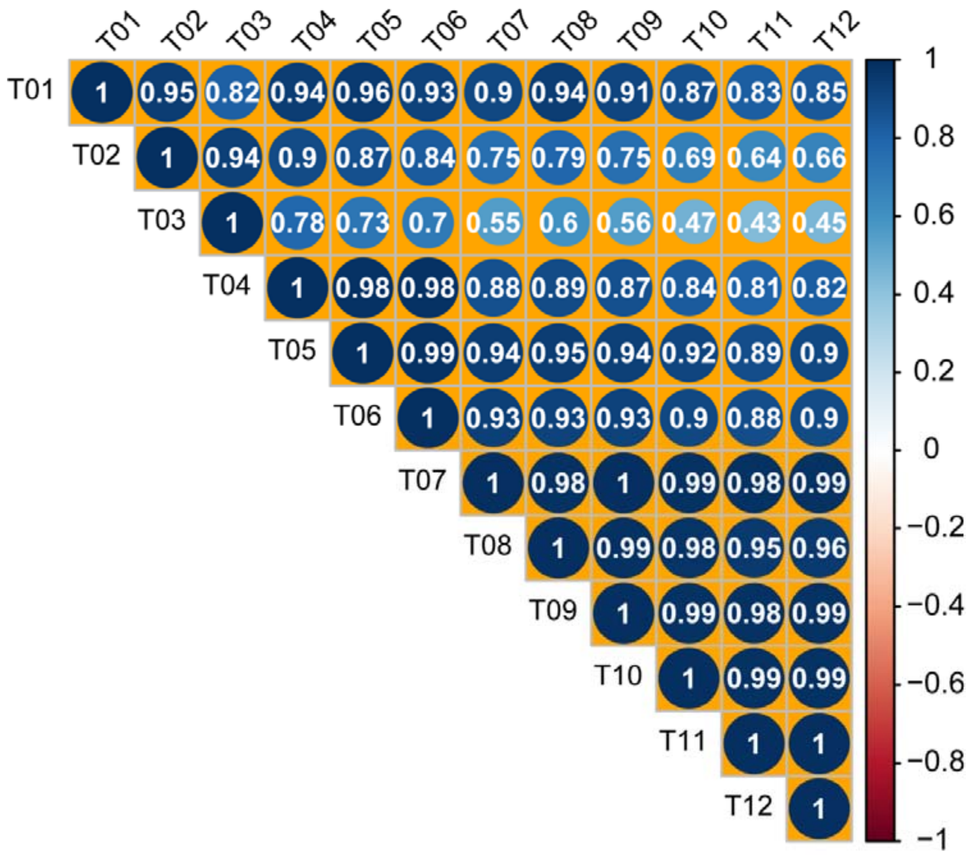

Supplementary Figure S1. Pearson's correlation matrix between biological replicates using cor R package. Three independent biological replicates for each bulbil developmental stages were used to construct RNA-seq libraries. Based on all gene expression data, Pearson correlation coefficients are highly correlated ($R^2 > 0.82$) between biological replicates, indicating that all collected samples for each stage were well processed.

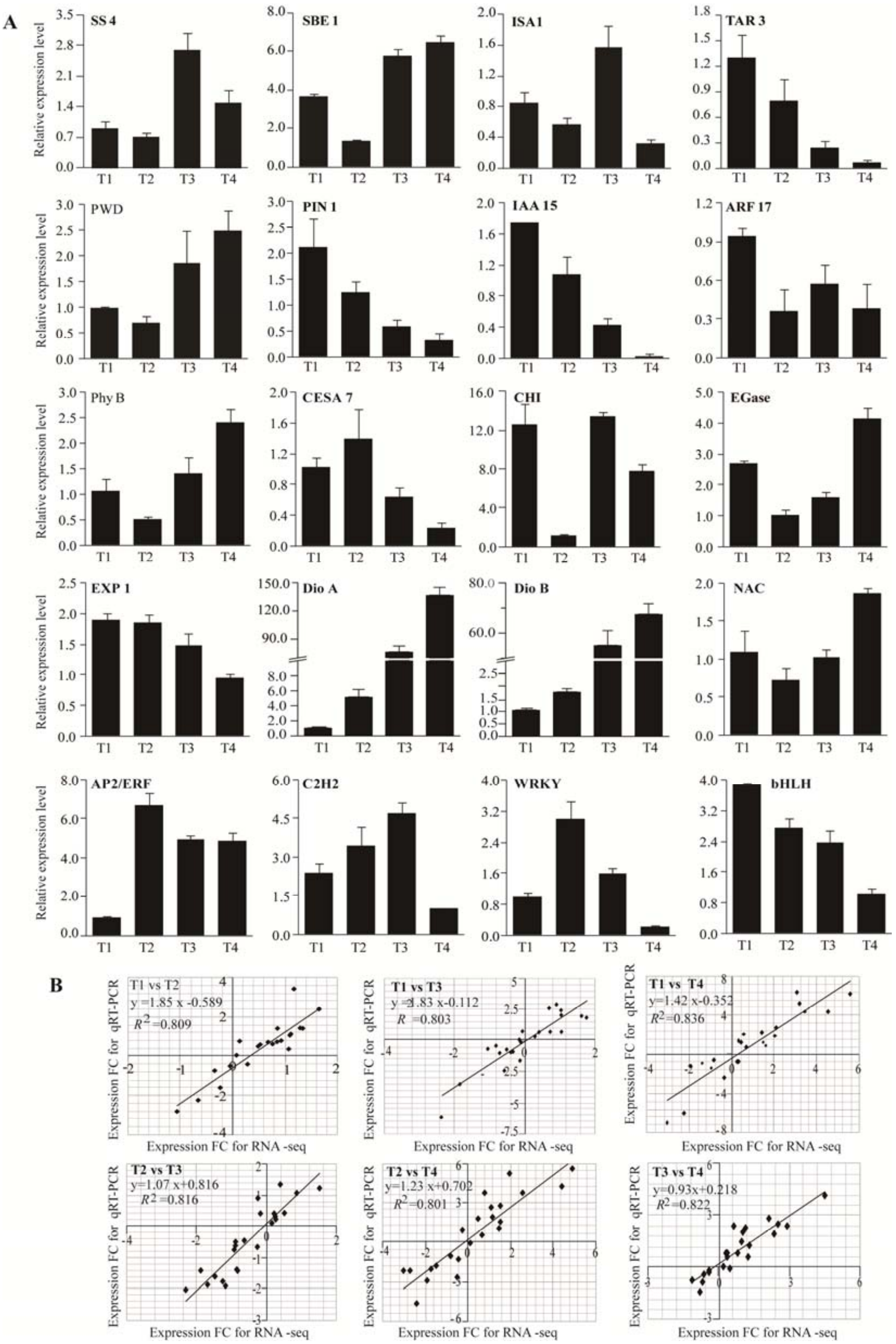

Supplementary Figure S2. Validations of gene expression profiles by qRT-PCR. **A,** Expression profiles for twenty selected DEGs were verified using qRT-PCR assay. Gene expression was normalized using EF-1a gene and quantified by the means of three independent qRT-PCR experiments ± SD. **B,** Scatter plot represents fold changes (T1vs T2, T1vsT3, T1vs T4, T2 vs T3, T2 vs T4 and T3 vs T4) in gene expressions measured by RNA-seq and qRT-PCR assay for selected

genes, indicating that the two technologies have a good agreement (R^2>0.8). ARF 17, auxin response factor 17; CESA 7; cellulose synthase 7; CHI, chitinase; Dio A, dioscorin A; Dio B, dioscorin B; EGase, Endo-1,3;1,4-beta-D-glucanase; EXP 1, EXPANSINS 1; IAA 15, auxin-responsive protein IAA15; ISA1, isoamylase 1; Phy B, phytochrome B; PIN1, auxin efflux carrier protein 1; PWD, phosphoglucan water dikinase; SBE 1, glucan-branching enzyme 1; SS4, starch synthase 4; TAR3, tryptophan aminotransferase 3.

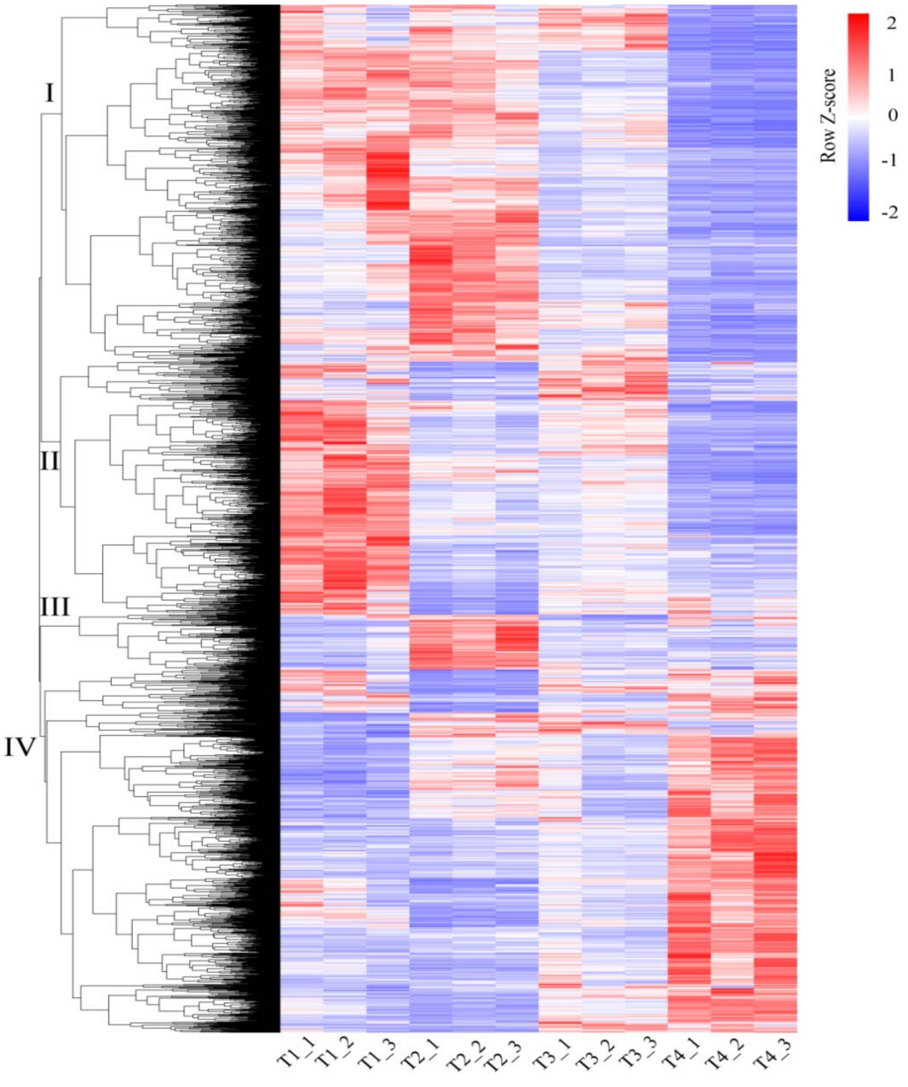

Supplementary Figure S3. Hierarchical clustering of all DEGs across different stages. Hierachical clustering seperate all DEGs into four groups, indicating four stage-specific expression patterns. Each row of the heat map represents an individual gene. The gene expression levels are standardized into Z-score and colored in red and blue for high and low expression.

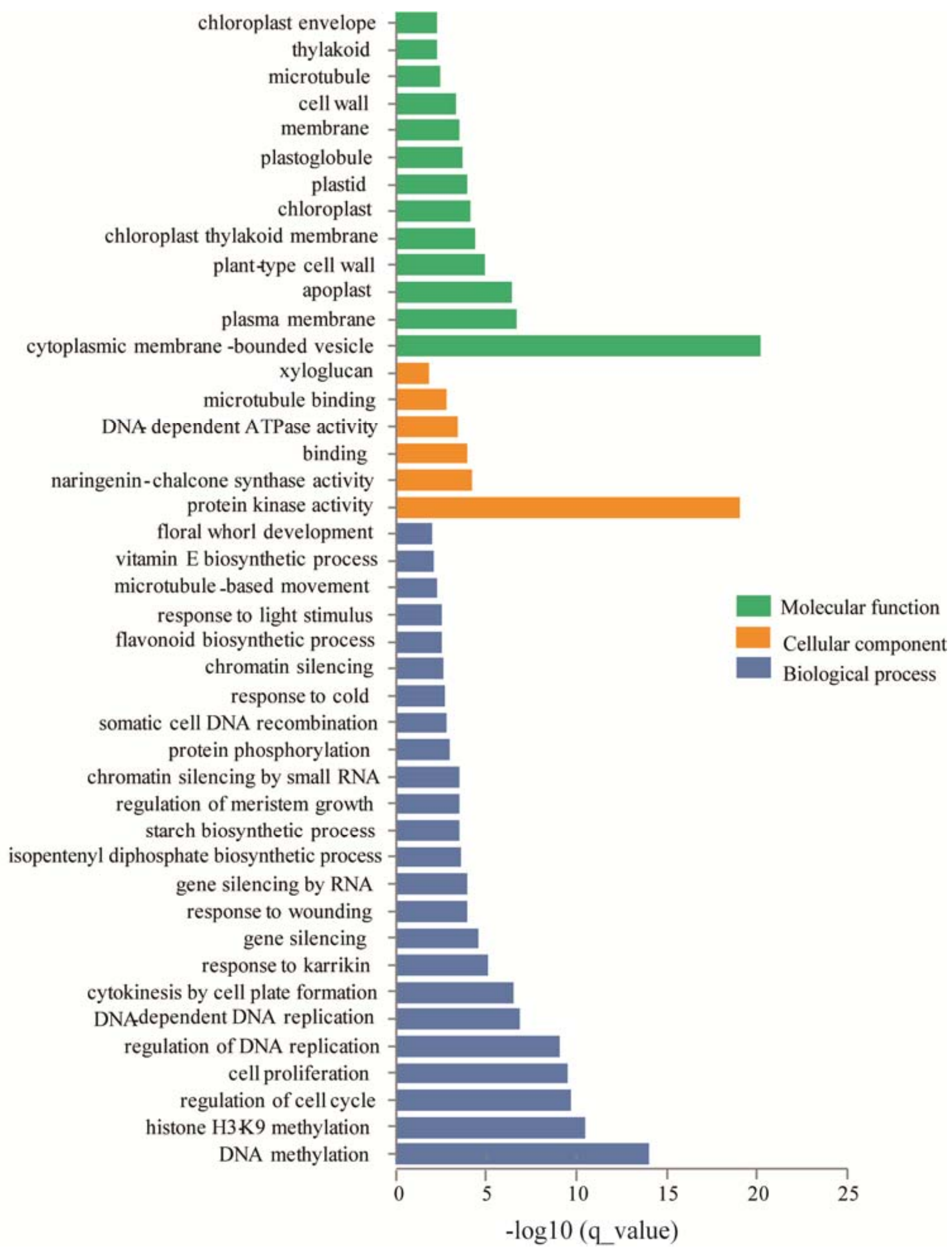

Supplementary Figure S4. The most enriched GO terms for all DEGs among level 1GO categories: biological process, molecular function, cellular component. GO enrichment are converted into negative logarithm score (base 10; values greater than 2.2 are denoted significant).

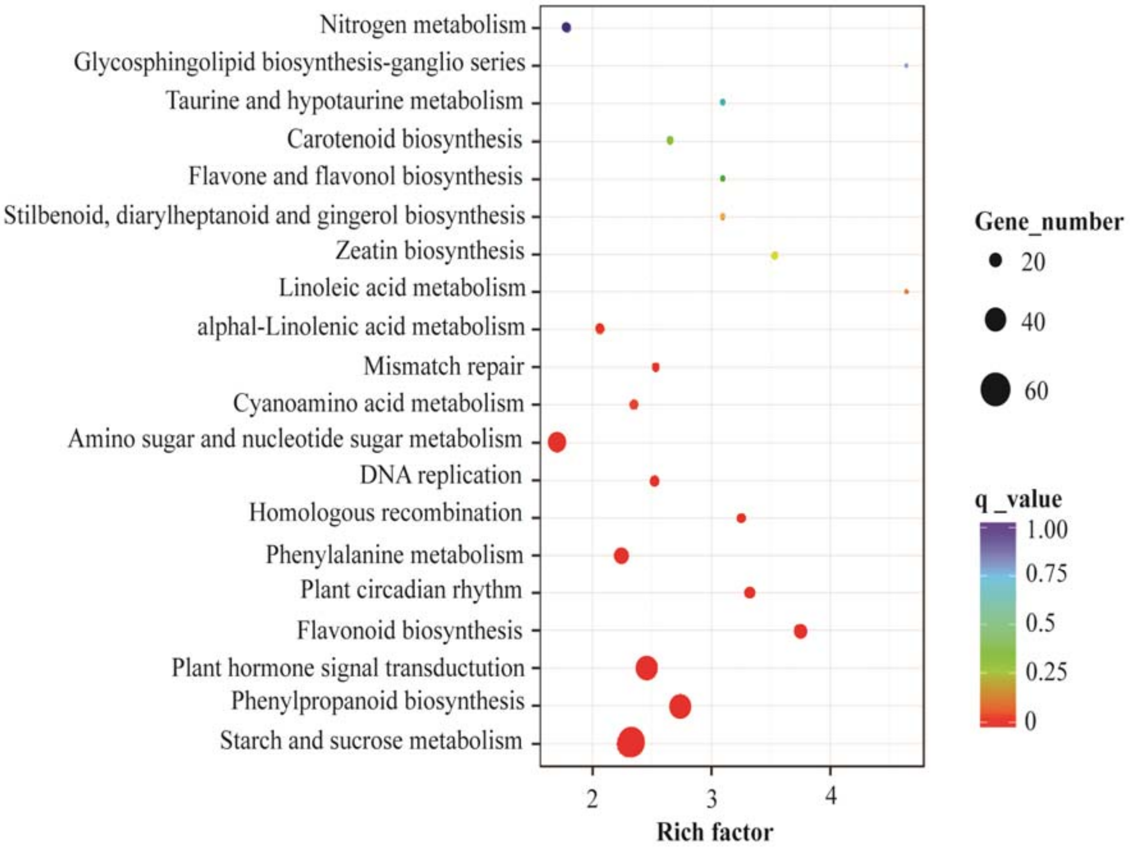

Supplementary Figure S5. Enriched KEGG pathways. All DEGs between different developmental stages were used to identify significantly enriched pathways using hypergeometric test with KOBAS2.0 software (http://kobas.cbi.pku.edu.cn/help.do). Columns and rows in this figure indicate enriched pathway terms and rich factor, respectively. The circle size represents the numbers of DEGs enriched in the specified KEGG pathway. Color code represents q-value (Corrected_P-value) of enrichment tests.

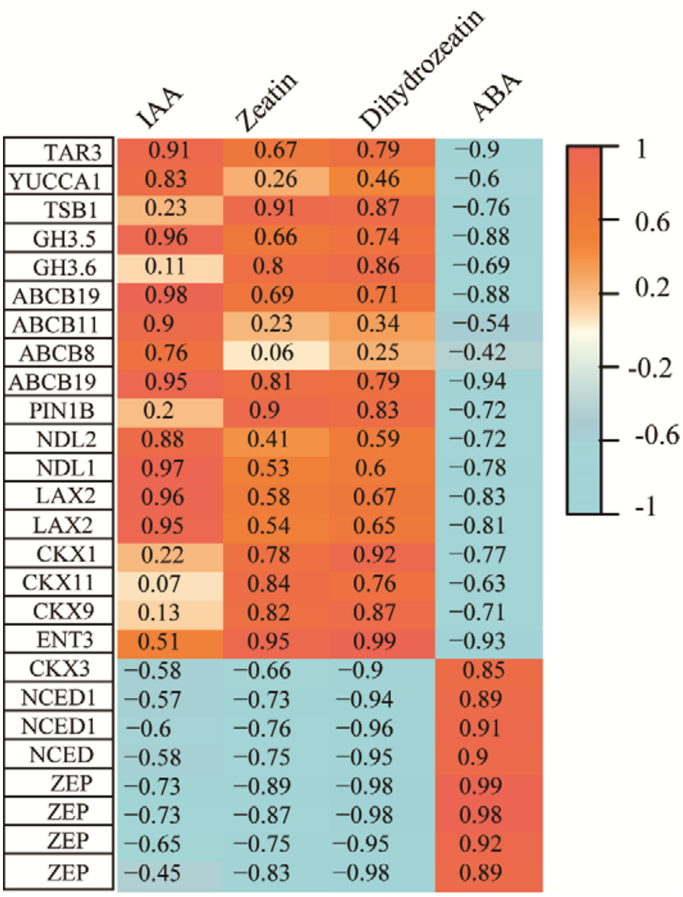

Supplementary Figure S6. Heat map of correlations between hormone (auxin, CKs and ABA) -related genes expression and its metabolite levels. The correlation is performed and visualized using cor R package. Gene names are described in Dataset S1 in detail.

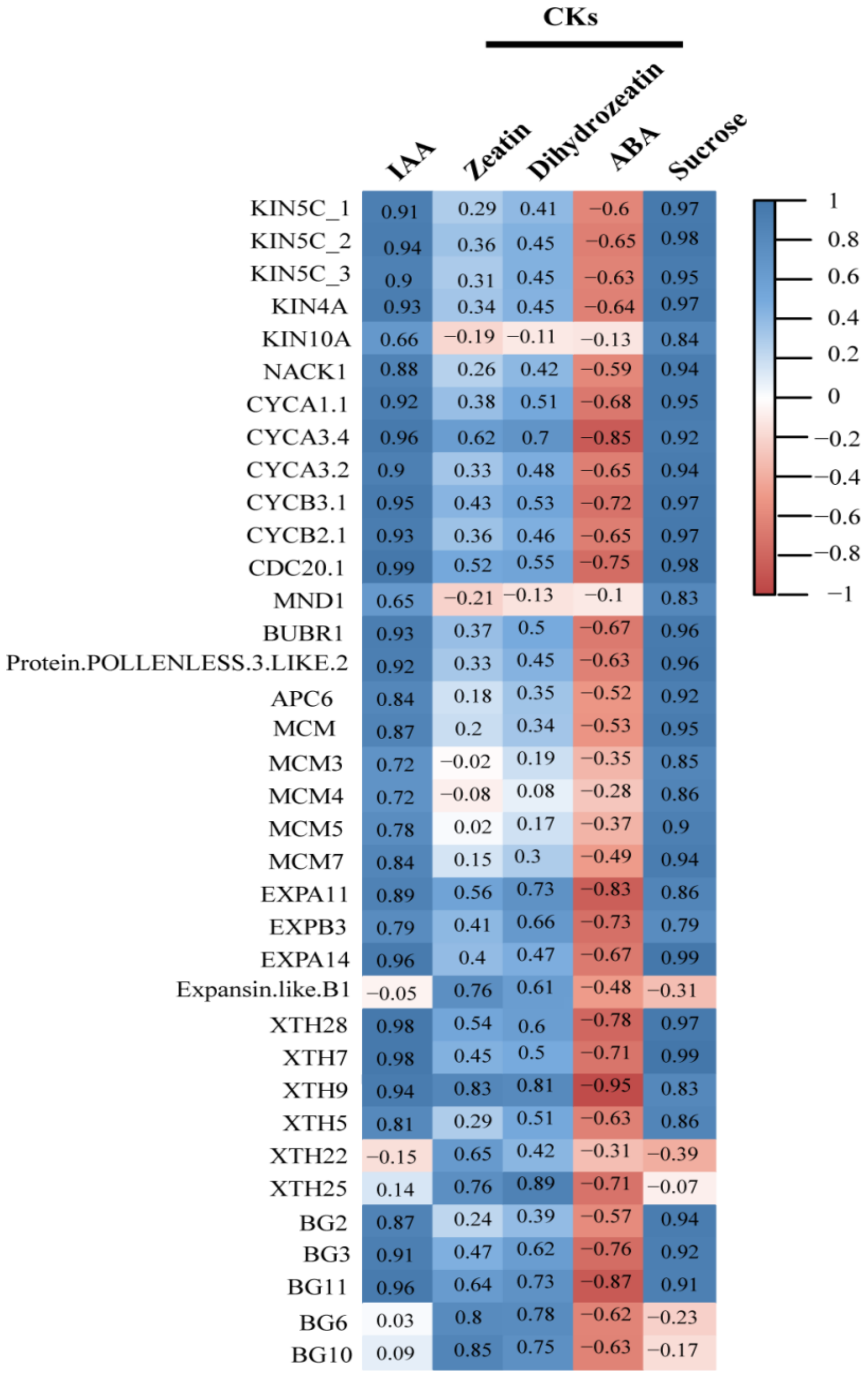

Supplementary Figure S7. Heat map of correlations between levels of hormone (auxin, CKs and ABA), sucrose and transcriptional expressions of genes associated with function categories: cell division, proliferation, cell wall expansion and modification, indicating positive correlations between auxin, CKs, sucrose and function categories investigated，and negative correlations between ABA and function categories investigated. These results demonstrate a potential evidence that auxin,CKS, ABA and sucrose (especially auxin and sucrose) act as integrated signals, and provide a forward forces to genes related to cell division, proliferation and expansion, promoting bulbil growth. The correlation is performed and visualized using cor R package. Gene names are described in Dataset S1 in detail.

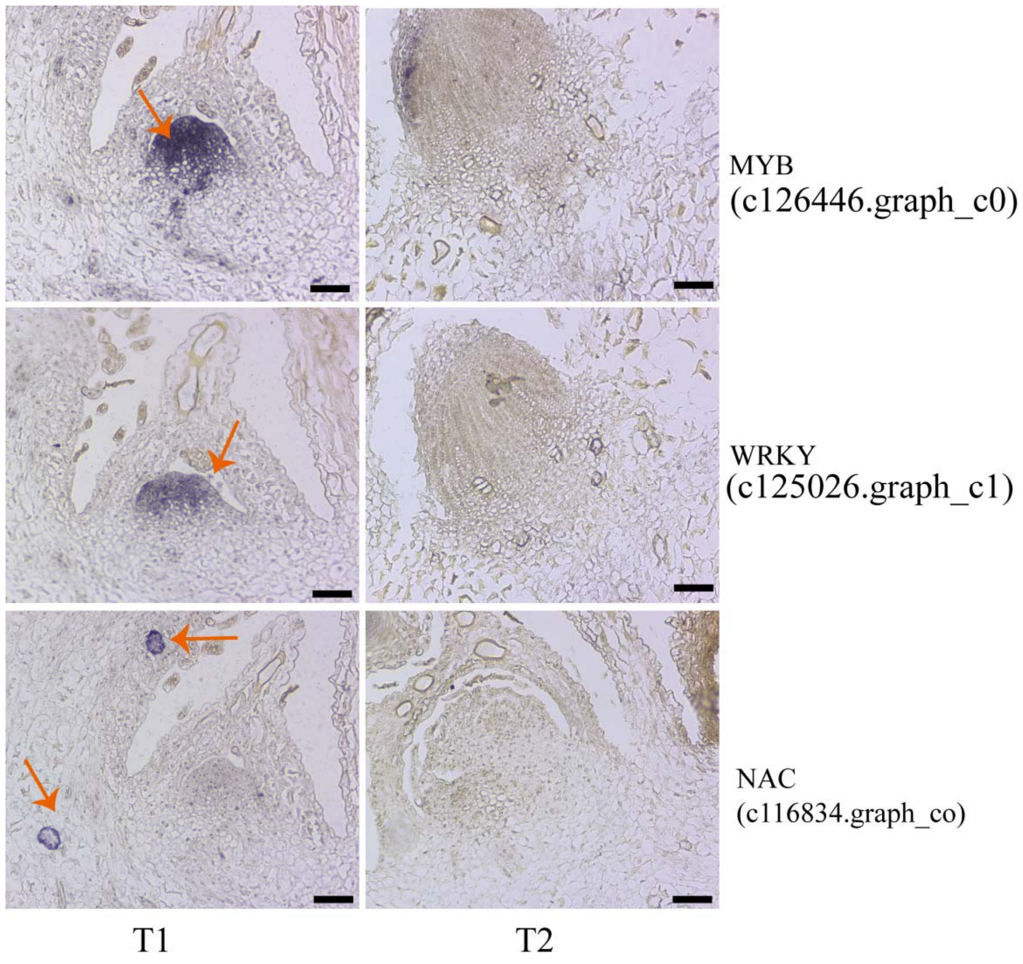

Supplementary Figure S8. RNA in situ hybridization for MYB, WRKY and NAC transcription factors. Three transcription factors show strongly accumulation in the AM initiation zone at the early stage of bulbil outgrowth (T1) (A, E, I). Bars=100 μm.
